# Cystine/glutamate antiporter xCT deficiency reduces metastasis without impairing immune system function in breast cancer mouse models

**DOI:** 10.1186/s13046-023-02830-x

**Published:** 2023-09-29

**Authors:** Roberto Ruiu, Chiara Cossu, Antonella Iacoviello, Laura Conti, Elisabetta Bolli, Luca Ponzone, Jolanda Magri, Alekya Rumandla, Enzo Calautti, Federica Cavallo

**Affiliations:** 1 Department of Molecular Biotechnology and Health Sciences, Laboratory of Oncoimmunology, Molecular Biotechnology Center “Guido Tarone”, University of Turin, Via Nizza 52, Turin, 10126 Italy; 2 Department of Molecular Biotechnology and Health Sciences, Laboratory of Epithelial Stem Cell Biology and Signaling, Molecular Biotechnology Center “Guido Tarone”, University of Turin, Via Nizza 52, Turin, 10126 Italy; 3 Present Address: Laboratory of Immunotherapy, IIGM - Italian Institute for Genomic Medicine, c/o IRCCS, Candiolo, Italy; 4 Present Address: Candiolo Cancer Institute, FPO - IRCCS, Candiolo, Italy; 5 Present Address: Biocon Bristol Myers Squibb R&D Center, Bommasandra Jigani Link Road, Bommasandra Industrial Area, Bangalore, Karnataka 560099 India

**Keywords:** xCT, SLC7A11, Immune system, Breast cancer, Metastasis, Metastatic niche

## Abstract

**Background:**

The upregulation of antioxidant mechanisms is a common occurrence in cancer cells, as they strive to maintain balanced redox state and prevent oxidative damage. This includes the upregulation of the cystine/glutamate antiporter xCT, which plays a crucial role in protecting cancer cells from oxidative stress. Consequently, targeting xCT has become an attractive strategy for cancer treatment. However, xCT is also expressed by several types of immune cells where it has a role in proliferation and effector functions. In light of these observations, a comprehensive understanding of the specific role of xCT in the initiation and progression of cancer, as well as its potential impact on the immune system within the tumor microenvironment and the anti-tumor response, require further investigation.

**Methods:**

We generated xCT^null^ BALB/c mice to investigate the role of xCT in the immune system and xCT^null^/*Erbb2*-transgenic BALB-neuT mice to study the role of xCT in a mammary cancer-prone model. We also used mammary cancer cells derived from BALB-neuT/xCT^null^ mice and xCT^KO^ 4T1 cells to test the contribution of xCT to malignant properties in vitro and in vivo.

**Results:**

xCT depletion in BALB-neuT/xCT^null^ mice does not alter autochthonous tumor initiation, but tumor cells isolated from these mice display proliferation and redox balance defects in vitro. Although xCT disruption sensitizes 4T1 cells to oxidative stress, it does not prevent transplantable tumor growth, but reduces cell migration in vitro and lung metastasis in vivo. This is accompanied by an altered immune cell recruitment in the pre-metastatic niche. Finally, systemic depletion of xCT in host mice does not affect transplantable tumor growth and metastasis nor impair the proper mounting of both humoral and cellular immune responses in vivo.

**Conclusions:**

xCT is dispensable for proper immune system function, thus supporting the safety of xCT targeting in oncology. Nevertheless, xCT is involved in several processes required for the metastatic seeding of mammary cancer cells, thus broadening the scope of xCT-targeting approaches.

**Supplementary Information:**

The online version contains supplementary material available at 10.1186/s13046-023-02830-x.

## Background

Cancer cells are characterized by altered metabolic programs in a challenging microenvironment, which can lead to excessive oxidative stress that can ultimately result in cancer cell death [1–3]. Therefore, cancer cells upregulate antioxidant mechanisms to maintain redox homeostasis [4].

Solute Carrier Family 7 member 11 (*SLC7A11*) is a key gene involved in redox regulation in cancer cells and codes for the multipass transmembrane protein xCT. xCT dimerizes with the transmembrane chaperone protein SLC3A2 to form the amino acid transport system xc^−^ [5]. While SLC3A2 allows localization of the transporter to the plasma membrane and acts as a subunit for several other transporter systems, xCT provides the substrate specificity [5].

System xc^−^ acts as a Na^+^-independent cystine/glutamate antiporter by importing cystine (but not cysteine) and exporting glutamate at a 1:1 ratio [5]. Once inside the cell, cystine is reduced to cysteine, which is the rate-limiting precursor for the biosynthesis of the antioxidant molecule glutathione (GSH) [5]. Therefore, xCT is responsible for scavenging of reactive oxygen species (ROS) by mediating the import of cystine into the cells, thereby preventing excessive oxidative stress and allowing cancer cell survival [6]. Other transporters import cysteine; however, cystine is more abundant than cysteine because of the strongly oxidizing extracellular environment [7]. Hence, cells rely on xCT to fulfill their cysteine needs by importing cystine.

Different tumor types, including breast cancer, overexpress xCT [8, 9] to sustain proliferation and resistance to ROS-inducing chemotherapy and radiotherapy [10, 11], and high xCT expression is associated with poor prognosis in various tumor types, including breast cancer [9, 12]. In addition, xCT targeting has detrimental effects on cancer cells, resulting in reduced tumor growth and metastasis in mice, and sensitization to chemotherapy [13]. Therefore, xCT represents a potential target for breast cancer treatment [8]. Sulfasalazine (SAS), Erastin, and Sorafenib [6] are pharmacological inhibitors of system xc^-^, and we recently developed several anti-xCT vaccine formulations [14].

However, an xCT-targeting strategy may affect not only cancer cells, but also cells of the immune system. Indeed, xCT is expressed by activated macrophages, granulocytes, and T cells [5, 15], where it supports their physiological functions. xCT was also found to be expressed by immunosuppressive cell populations, such as Myeloid-Derived Suppressor Cells (MDSC) [16] and regulatory T cells (Treg) [17]. It was recently shown that a systemic lack of xCT preserves adaptive antitumor immunity [18], but a full understanding of the effects of xCT modulation in the immune system is still lacking. In addition, many studies to date have focused on the cell-autonomous functions of xCT in immunocompromised mouse models, thus neglecting the role of xCT in the interaction of cancer cells with the surrounding microenvironment and in tumor-related immune responses.

The expression of xCT in cancer and immune cells could have non-cell-autonomous effects, in addition to its cell-autonomous role in redox balance maintenance. xCT-mediated secretion of glutamate by tumor cells promotes Treg activation and their immunosuppressive functions [19], while MDSC compete with antigen-presenting cells for importing cystine through xCT, hindering T cell activation [16]. Moreover, xCT is involved in the release of extracellular vesicles (EV) by tumor cells [20–22], which play a role in communication with various components of the microenvironment [23].

Finally, although the contribution of xCT to the malignant features of mammary cancer has been reported in the literature, no prior research has investigated its role in tumor initiation and progression in mammary-cancer-prone models.

Hence, the goal of this study was to address these currently neglected aspects of xCT contribution to the development of mammary cancer. On one hand, we investigated for the first time the role of xCT in mammary tumor initiation using a mouse model that lacks xCT and is predisposed to developing mammary cancer (BALB-neuT/xCT^null^). On the other hand, we elucidated the differential effects of xCT on both the malignancy of tumor cells and the immune response to tumors by utilizing xCT-proficient and xCT-deficient mammary cancer cells and host mice, respectively. Moreover, we here provide evidence supporting the notion that xCT has non-cell-autonomous functions. These findings may broaden the scope of the currently tested therapeutic approaches against xCT.

## Methods

### Cell lines

4T1 (ATCC Cat# CRL-2539) cells were purchased from ATCC and cultured in RPMI-1640 medium (Sigma-Aldrich) supplemented with 10% Fetal Bovine Serum (FBS, Sigma-Aldrich). The growth medium of the 4T1 xCT^wt^ and xCT^KO^ clones was further supplemented with 100 µM β-Mercaptoethanol (β-ME; Sigma-Aldrich), while the growth medium of 4T1-pLVX and of xCT-overexpressing 4T1 cells (either derived from parental cells or xCT^KO^ pool) was supplemented with 3 µg/mL puromycin (Sigma-Aldrich). SUT32-2H9 and WT27 cells were cultured in DMEM-F12 medium (Sigma-Aldrich) containing 20% FBS and 100 µM β-ME. The growth medium of SUT32-2H9-pLVX and SUT32-2H9-xCT was further supplemented with 1.5 µg/mL puromycin. Where indicated, SUT32-2H9 cells were cultured in Human Plasma-Like Medium (HPLM, Gibco) supplemented with 20% dialyzed FBS. All growth media were supplemented with penicillin/streptomycin (P/S) solution (Sigma-Aldrich). Cells were maintained in humidified incubators at 37 °C with 5% CO_2_. The cells were periodically tested for mycoplasma contamination using the MycoAlert™ Mycoplasma Detection Kit (Lonza Cat# LT07-318) according to the manufacturer’s instructions. All cells used were free of mycoplasma. All cells were used within 10 passages from thawing and were kept in culture cumulatively for no more than six months. All cell lines used originated from female mice. Further details on the establishment of SUT32-2H9 and WT27 cell lines from tumors of BALB-neuT/xCT^null^ and xCT^wt^ mice, as well as on selenocystine uptake assay, MTT assay, colony forming efficiency assay, ROS and lipid peroxidation detection, and migration assays can be found in the Additional file 2, Supplementary Methods section.

### Generation of xCT^KO^ 4T1 cells and of xCT-overexpressing cells

4T1 cells were transfected using Lipofectamine 2000 (Thermo Fisher Scientific Cat# 11668-019) according to the manufacturer’s instructions, with either an empty (px459) or *Slc7a11*-targeting (px459-xCT) CRISPR/Cas9 vector (details on vector design and production are reported in the Additional file 2, Supplementary Methods section). The following day, the transfection medium was replaced with RPMI-1640 medium supplemented with 10% FBS and 100 µM β-ME. Puromycin was added at a final concentration of 3.5 µg/mL. After 48 h selection, cells were plated at 0.3 cells/well in 96-well plates, and single-cell clones were expanded further. The lack of xCT expression was confirmed by western blot analysis. To generate xCT-overexpressing cell lines, SUT32-2H9 and 4T1 target cells were stably transduced with a lentiviral vector expressing the coding sequence of murine xCT under the control of the CMV promoter. Further details are provided in the Additional file 2, Supplementary Methods section.

### Western blot

Cells were incubated with RIPA lysis buffer supplemented with 1 mM phenylmethylsulfonyl fluoride, 1 mM NaVO_4_, 1 mM NaF, and a protease inhibitor cocktail (Sigma-Aldrich) on ice. Proteins (20–30 µg) were added to Laemmli Buffer (Bio-Rad Laboratories) supplemented with 2.5% β-ME and left at room temperature for 30 min. Proteins were separated using a 4–15% polyacrylamide Precast Gel (Bio-Rad Laboratories) and transferred to a PVDF membrane (Immobilion-P, 0.45 μm pore size, Merck Millipore). In some instances, stain-free gels (Bio-Rad Laboratoires) were used. Non-specific binding sites were blocked using 5% non-fat milk (Santa Cruz Biotechnology) in T-TBS 1X (Tris Buffered Saline, 0.1% Tween 20). Membranes were incubated with mouse anti-vinculin (produced in-house, 1:8000) or rabbit anti-mouse xCT (#98051, Cell Signaling Technology; 1:1000), followed by incubation with HRP-linked goat anti-mouse (Sigma-Aldrich, Cat# A0545; 1:2000) or HRP-linked goat anti-rabbit IgG (Sigma-Aldrich, Cat# A4416; 1:2000), respectively. ECL substrate (Cyanagen Cat# XLS142,0250) was used for signal detection, and images were acquired using a Chemidoc™ Touch Imaging System (Bio-Rad Laboratories).

### Mouse models

BALB/c and BALB-neuT mice [24] were obtained from an internal breeding. C3H/HeSnJ-*Slc7a11*^*sut*^/J mice [25] were purchased from The Jackson Laboratory and crossed with BALB/c and BALB-neuT mice to obtain BALB/c-xCT^null^ and BALB-neuT/xCT^null^ mice, respectively. The details of the crossing and genotyping are reported in the Additional file 2, Supplementary Methods section. Animals were group housed, and food and water were provided *ad libitum*. All mice were maintained at the Molecular Biotechnology Center, University of Turin.

### Tumor monitoring

10-12-week-old BALB/c-xCT^wt^ or BALB/c-xCT^null^ females were challenged subcutaneously (s.c.) with cancer cells in the left flank region, corresponding to the 4^th^ mammary gland. Number of cells injected is reported in the corresponding figure legend. Tumor growth was monitored twice a week using a caliper, and the experimental endpoint was set at 28–33 days after injection (or at days 8 and 15 after injection, where specified). In BALB-neuT and BALB-neuT/xCT^null^ mice, tumor onset and growth were monitored weekly, and the endpoint was set according to ethical criteria. For intravenous (i.v.) cancer cell injection, 10.000 cells (parental 4T1 and pool of 4T1 xCT^KO^ clones) were suspended in 100 µL PBS and injected into the caudal vein of female BALB/c mice, which were culled 28 days post-injection.

### Immunophenotyping

At the experimental endpoint, the mice were anesthetized, and peripheral blood was collected through intracardiac puncture and supplemented with heparin solution. The mice were euthanized, and the lungs, tumor, and spleen were collected. The left lung and the tumor were finely minced and enzymatically digested with 100 µg/mL collagenase in DMEM at 37 °C for 30 min and 45 min, respectively, under shaking. The digested tissues were passed through a 70 μm pore cell strainer to obtain a single-cell suspension. Erythrocytes were lysed using an erythrocyte lysis buffer. An Fc Blocking antibody (anti-CD16/CD32 antibody, BioLegend Cat# 101,320) was added, and the cells were stained at 4 °C for 30 min with fluorochrome-labeled antibodies. Samples were acquired using a BD FACSVerse™ instrument, and data were analyzed using FlowJo V10 software. Details of the staining procedure and antibodies used, as well as the phenotyping protocol of SUT32-2H9 and WT27 cell lines, can be found in the Additional file 2, Supplementary Methods section.

### In vitro polarization of bone marrow-derived cells

Mice were euthanized and femurs and tibias were collected and stored in ice-cold RPMI-1640 supplemented with 10% FBS and 1% P/S. Bone marrow (BM) was extracted as previously described [26]. BM cells were incubated in erythrocyte lysis buffer, rinsed with PBS, pelleted, and seeded in a 6 well-plate in RPMI-1640 with 10% FBS or supernatant from 72 h-cultured 4T1 cells. The cells were incubated at 37 °C and replenished with fresh or conditioned medium (CM) every second day. On day 7, the suspension and adherent cells were pooled and stained with fluorochrome-labeled antibody combinations for macrophages/myeloid cells, DC, and B cells, as described in the extended immunophenotyping section (Additional file 2, Supplementary Methods) and were acquired and analyzed as described above.

### DNA-based vaccination and SAS treatment

Mice were anesthetized and vaccinated with two intramuscular injections of 20 µL of physiological solution containing 50 µg of pVAX1 (Thermo Fisher Scientific Cat# V26020) or RHuT [27] plasmids followed by low-voltage electroporation as previously described [27], at a 12–14 day interval. When combined with SAS treatment, the first vaccination was performed 4 days after the beginning of the treatment, while the second vaccination preceded of 4 days the end of the treatment (Supplementary Fig. S2A). Each mouse received an intraperitoneal injection of 4 mg of SAS (Sigma-Aldrich, Cat# S0883) in 400 µL of saline solution, twice daily, for 22 consecutive days (Supplementary Fig. S2A). This resulted in a total daily dose of 8 mg of SAS per mouse, approximately equivalent to 400 mg/kg of body weight. The SAS solution was freshly prepared prior to administration. Initially, SAS was suspended in a small volume of 1 N NaOH (200 mg/mL), then diluted with saline solution and adjusted to a pH of around 8 using 1 N HCl. Subsequently, the SAS solution was further adjusted to a final concentration of 10 mg/mL using saline solution.

### In vivo cytotoxicity assay

Spleens from donor mice were disaggregated using a syringe plunger over a 70 μm cell strainer, incubated in erythrocyte lysis buffer, rinsed in PBS, and centrifuged. Equal amounts of cells were incubated at a final concentration of either 5 (CFSE^high^) or 0.5 (CFSE^low^) µM carboxyfluorescein succinimidyl ester (CFSE; Thermo Fisher Scientific Cat# V12883). CFSE^high^ splenocytes were then incubated with a final concentration of 15 µg/mL immunodominant rat ERBB2 (p185neu [63–71] 9-mer) peptide with H-2K^d^ restriction. Equal amounts of CFSE^high^ and CFSE^low^ splenocytes were mixed at a 1:1 ratio and injected intravenously into pVAX1- or RHuT-vaccinated mice. After 48 h, the recipient mice were euthanized, spleens were collected and disaggregated as described above, and the proportion of CFSE^high^ and CFSE^low^ splenocytes was assessed using FACS.

### Rat and human ERBB2 ELISA

96-well plates (Costar) were coated with 100 ng/well of recombinant extracellular portions of rat (Sino Biological Cat# 80,079-R08H, His Tag) or human (Sino Biological Cat# 10,004-H08H, His Tag) ERBB2 proteins. ELISA of sera from vaccinated mice to detect anti-ERBB2 IgG was then carried out as previously described [28].

### Statistical analysis

Unless otherwise specified, an unpaired t test was used to assess statistically significant differences between the groups. Ratio paired t test was used to assess consistency in the ratios of paired values (where values of experimental groups were normalized on values of control groups), Fisher’s test was used to assess significant differences in the incidence of lung metastases, and Log-Rank (Mantel-Cox) test was used to assess differences in the disease-free survival of BALB-neuT and BALB-neuT/xCT^null^ mice. Statistical analysis was performed using the GraphPad Prism v8 software. *p* < 0.05 was considered significant. Definition of center and of dispersion and precision measures (e.g., mean and SD), as well as the number of technical or biological replicates of the experiments described and the specific statistical test used, are reported in the corresponding figure legends. For in vivo experiments, mice were assigned to a given experimental group via simple randomization. Experimenters were not blind to group assignment and outcome assessment.

## Results

### Initiation of autochthonous mammary tumors in cancer-prone mice is not dependent on xCT

**Fig. 1:**
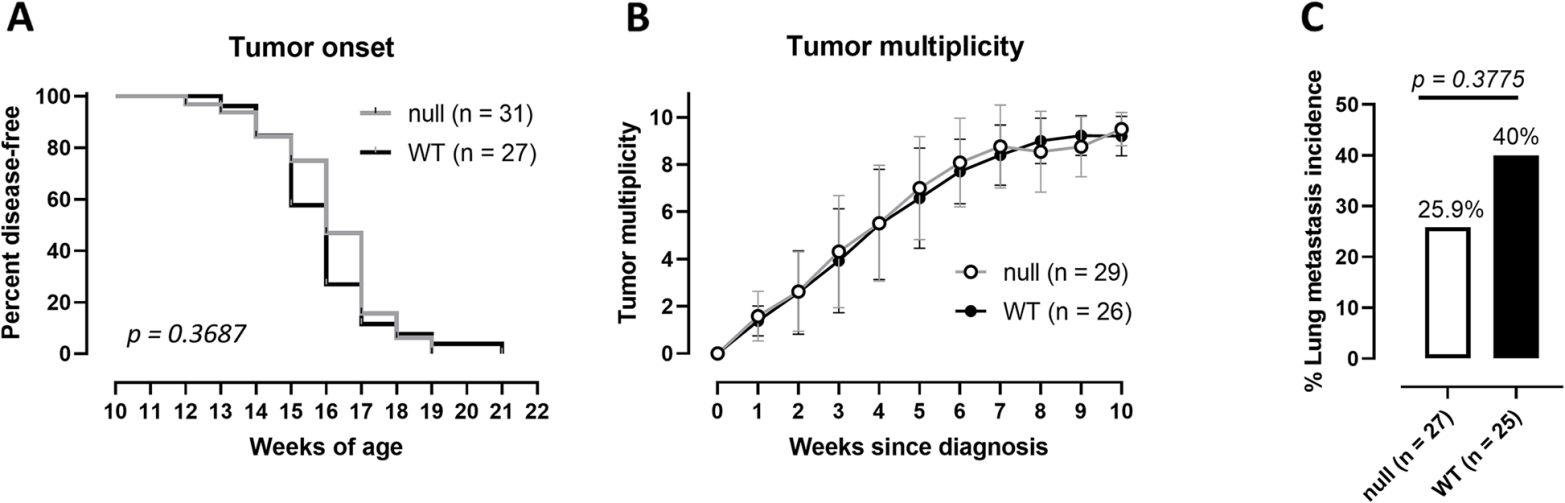
Initiation of autochthonous mammary tumors in cancer-prone mice is not dependent on xCT*. ***A** Percentage of disease free-survival and **B** tumor multiplicity from disease onset in BALB-neuT/xCT^wt^ (WT) and BALB-neuT/xCT^null^ (null) mice. **C** Percentage of mice affected by lung metastasis at sacrifice. Numbers of mice are reported in the panel legends. Statistical analysis: Log-rank (Mantel-Cox) test (panel A), unpaired t test (panel B), or Fisher’s exact test (panel C). Where not indicated, *p* value is not significant. In some instances, *p* values are represented in numbers when not significant. In panel B, mean values ± SD are depicted

To assess the contribution of xCT to mammary tumor initiation, mammary cancer-prone mice (BALB-neuT) [29] and xCT^null^ mice (BALB/c-*Slc7a11*^*sut*^) [25, 30] were bred to obtain BALB-neuT/xCT^null^ mice. The lack of xCT in these mice did not alter mammary tumor latency (Fig. 1A) or multiplicity (Fig. 1B) compared to BALB-neuT/xCT^wt^. Nevertheless, BALB-neuT/xCT^null^ mice showed a trend of reduction in the incidence of lung metastasis (Fig. 1C) when culled for ethical reasons at 20–25 weeks of age (Supplementary Fig. S1A). Although xCT is naturally expressed in cells of the immune system, no difference between BALB-neuT/xCT^wt^ and BALB-neuT/xCT^null^ mice was observed in the immune subpopulations in the blood (Supplementary Fig. S1B), lungs (Supplementary Fig. S1C), and tumors (Supplementary Fig. S1D). Additionally, spleen weight did not differ significantly between the two groups of mice (Supplementary Fig. S1E), nor did the degree of immune infiltration within the tumor (Supplementary Fig. S1F). Overall, the systemic lack of xCT did not affect either autochthonous tumor initiation or accompanying alterations in immune cell proportions.

### Immune response is preserved in xCT^null^ mice

**Fig. 2:**
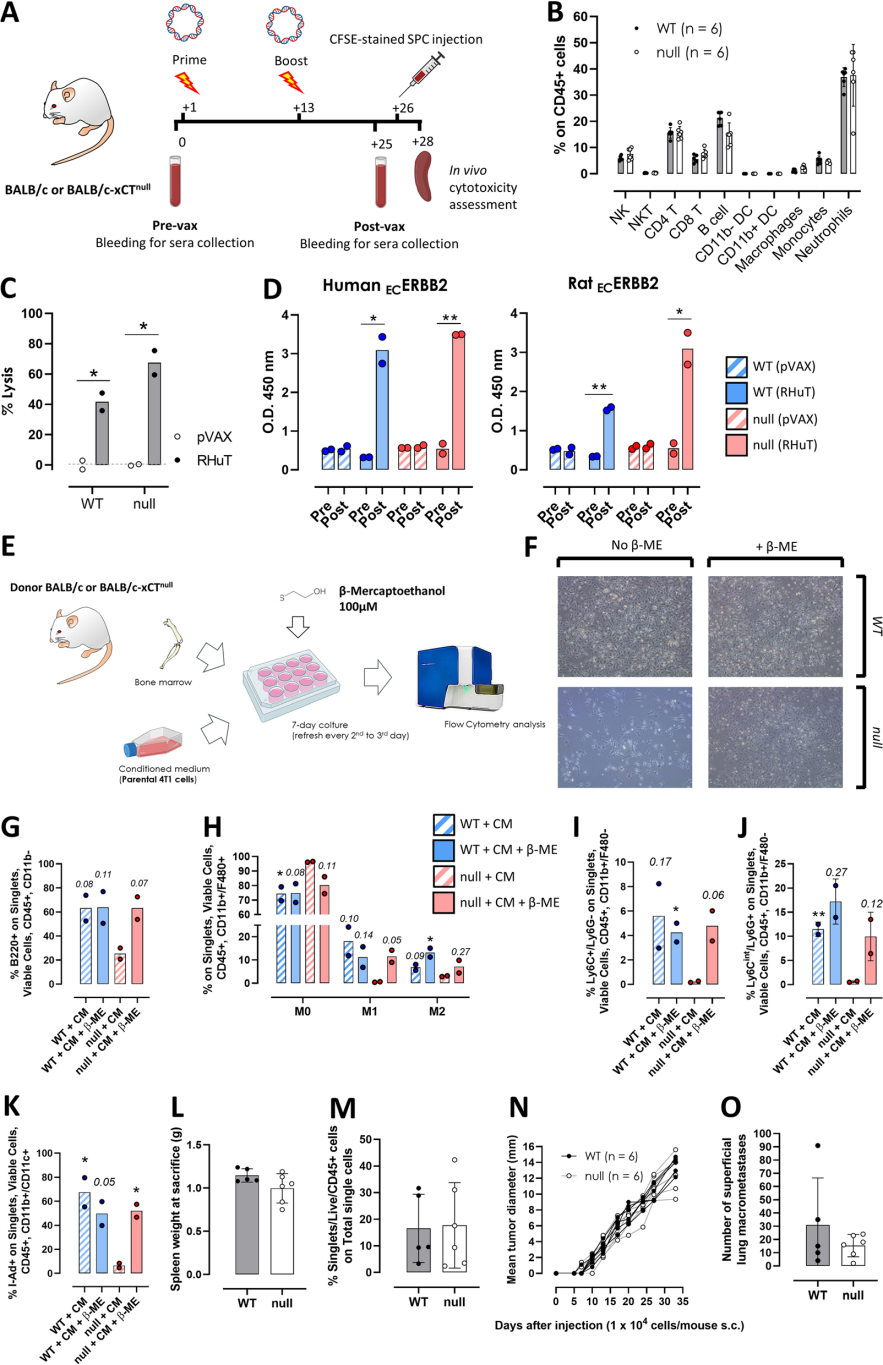
Immune response is preserved in xCT^null^ mice. **A** Immunization schedule of xCT^wt^ (WT) and xCT^null^ (null) BALB/c mice with the empty pVAX1 or the RHuT plasmids. **B** Percentage of immune cell populations in the blood of WT and null healthy mice. **C**  Percent of lysis of splenocytes stained with CFSE, pulsed with the target antigen, and injected in mice immunized with pVAX1 or RHuT, as assessed by flow cytometry. **D** ELISA assay of pre-vax (Pre) and post-vax (Post) sera from vaccinated mice, recognizing Human or Rat ERBB2 extracellular (EC) portion. **E** Experimental scheme of BM-derived cells culturing and conditioning. **F** Brightfield microscopy images (10X) of WT or null BM-derived cells cultured for 7 days with CM of 4T1 cells, with or without the addition of 100 µM β-ME. Percentage of **G** B lymphocytes, **H** polarized macrophages, **I** monocytes/mMDSC, **J** neutrophils/PMN-MDSC and **K** mature dendritic cells on their respective parent populations indicated in the y-axis legend. **L** Histogram representation of spleen weight at sacrifice of mice challenged subcutaneously with 10.000 4T1 cells. **M** Percentage of CD45+ tumor infiltrating cells on total single cells of mice described in panel L. **N** Growth curves of tumors deriving from 10.000 4T1 cells injected subcutaneously in WT or null mice. **O** Number of superficial metastases in lungs from mice described in panel N. Number of replicates: Each dot represents a mouse. Statistical analysis: unpaired t test. In panels G-K, t test was performed between xCT^null^(null) cells grown in CM without β-ME versus all the other groups. Unpaired t test performed between pre-vax and post-vax values, or between pVAX and RHuT values. * *p*<0.05; ** *p*<0.01. Where not indicated, *p* value is not significant. In some instances, *p* values are represented in number when not significant. Histograms represent mean values. Error bars (SD) are shown only when n ≥ 5

As we did not observe any difference in tumor initiation and growth in BALB-neuT/xCT^null^ mice compared to BALB-neuT/xCT^wt^ mice, we questioned whether any delay in tumor onset and progression in xCT^null^ mice could be masked by defective immunosurveillance due to a detrimental effect of the systemic lack of xCT on the immune system. To this end, we vaccinated both BALB/c-xCT^null^ and BALB/c-xCT^wt^ mice with a plasmid encoding a rat/human chimeric form of ERBB2 (RHuT vaccine), a xenogeneic antigen for BALB/c mice [31]. pVAX1 empty vector was used as control (Fig. 2A). We did not observe any difference in the proportions of immune populations in the blood before vaccination (Fig. 2B). In addition, the cytotoxic T cell response was preserved in BALB/c-xCT^null^ mice, since RHuT-vaccinated mice from both groups showed a comparable percentage of lysis in vivo of CFSE-stained splenocytes (SPC) pulsed with the immunodominant MHC-I-restricted epitope of rat ERBB2 (Fig. 2C). Similarly, the antibody response was preserved (Fig. 2D), since sera from both BALB/c-xCT^null^- and BALB/c-xCT^wt^-vaccinated mice contained antibodies recognizing human and rat ERBB2. Overall, these data show that systemic depletion of xCT does not impair the proper mounting of humoral and cellular immune responses in vivo upon vaccination with a xenogeneic antigen, indicating that the adaptive immune response is preserved even in the absence of xCT.

As the systemic lack of xCT since embryo development phases could potentially influence animal physiology, adaptation, and compensatory mechanisms, we opted for a pharmacological inhibition approach to provide more translatable evidence that targeting xCT in naturally expressing immune cells would not disrupt their normal functions. We treated BALB/c-xCT^wt^ mice with the clinically approved xCT inhibitor SAS, using a dosage of SAS that has demonstrated antitumor effects in preclinical testing [32]. The drug was administered twice daily for 22 consecutive days at a dose of 8 mg per mouse per day, roughly equivalent to 400 mg/kg. Concurrently, we treated BALB/c-xCT^null^ mice to account for potential off-target effects. Saline-treated control groups were included for both strains. During the saline or SAS treatment period, mice were vaccinated with either the control plasmid pVAX1 or the RHuT vaccine (Supplementary Fig. S2A) to assess whether acute xCT inhibition impairs humoral and cellular immune responses. Our data indicate that in vivo xCT inhibition using SAS does not hinder the development of humoral and cellular responses compared to saline-treated mice (assessed through ELISA and in vivo cytotoxicity assays) (Supplementary Fig. S2B, C). Additionally, no genotype-related differences in the immune response to vaccination were observed (Supplementary Fig. S2B, C), implying that the drug does not exert detrimental effects on the immune system and supporting our conclusion that genetic xCT deficiency does not impede the immune response. Furthermore, monitoring the mice’s weight throughout the treatment period revealed no drug-related toxicity in any group (Supplementary Fig. S2D). In summary, based on these findings, we can conclude that neither genetic xCT deficiency nor pharmacological xCT inhibition adversely affects the physiological adaptive immune response.

To determine whether immune cell progenitors of xCT^null^ mice show alterations in proliferation, differentiation, or polarization in response to stimuli from cancer cells, BM cells were harvested and cultured with CM from 4T1 murine mammary cells, which are known to release abundant amounts of cytokines [33] (Fig. 2E). BM cells cultured in a medium not previously conditioned by cancer cells did not proliferate. xCT^null^ BM cells cultured in CM showed a defect in proliferation compared to xCT^wt^ cells, which instead reached confluence. The addition of β-ME to 4T1-CM completely rescued this phenotype (Fig. 2F). The lack of xCT in BM cells impaired B cell proliferation (Fig. 2G), completely prevented both M1 and M2 macrophage polarization (Fig. 2H), and strongly reduced the percentage of monocytes/mMDSC, neutrophils/PMN-MDSC (Fig. 2I, J), and mature dendritic cells (Fig. 2K). The 4T1-CM supplemented with β-ME restored the differentiation and polarization of immune cell subpopulations.

We then investigated whether such differences could be replicated in vivo upon challenge of naïve BALB/c-xCT^wt^ and BALB/c-xCT^null^ mice with 4T1 cells. However, no differences were observed between the two groups of mice after subcutaneous injection of 4T1 cells in terms of spleen weight (Fig. 2L) and degree of immune infiltration within the tumor (Fig. 2M). In addition, percentages of immune cell populations in blood (Supplementary Fig. S2E), metastatic lungs (Supplementary Fig. S2F), and primary tumors (Supplementary Fig. S2G) remained unchanged. Accordingly, a systemic lack of xCT did not affect primary tumor progression (Fig. 2N) nor the development of lung metastasis (Fig. 2O). Overall, these results suggest that xCT is required for proper immune system functionality in vitro but is dispensable in vivo likely because of compensatory mechanisms.

### xCT depletion in cancer cells affects response to oxidative stress and cell migration in vitro

**Fig. 3:**
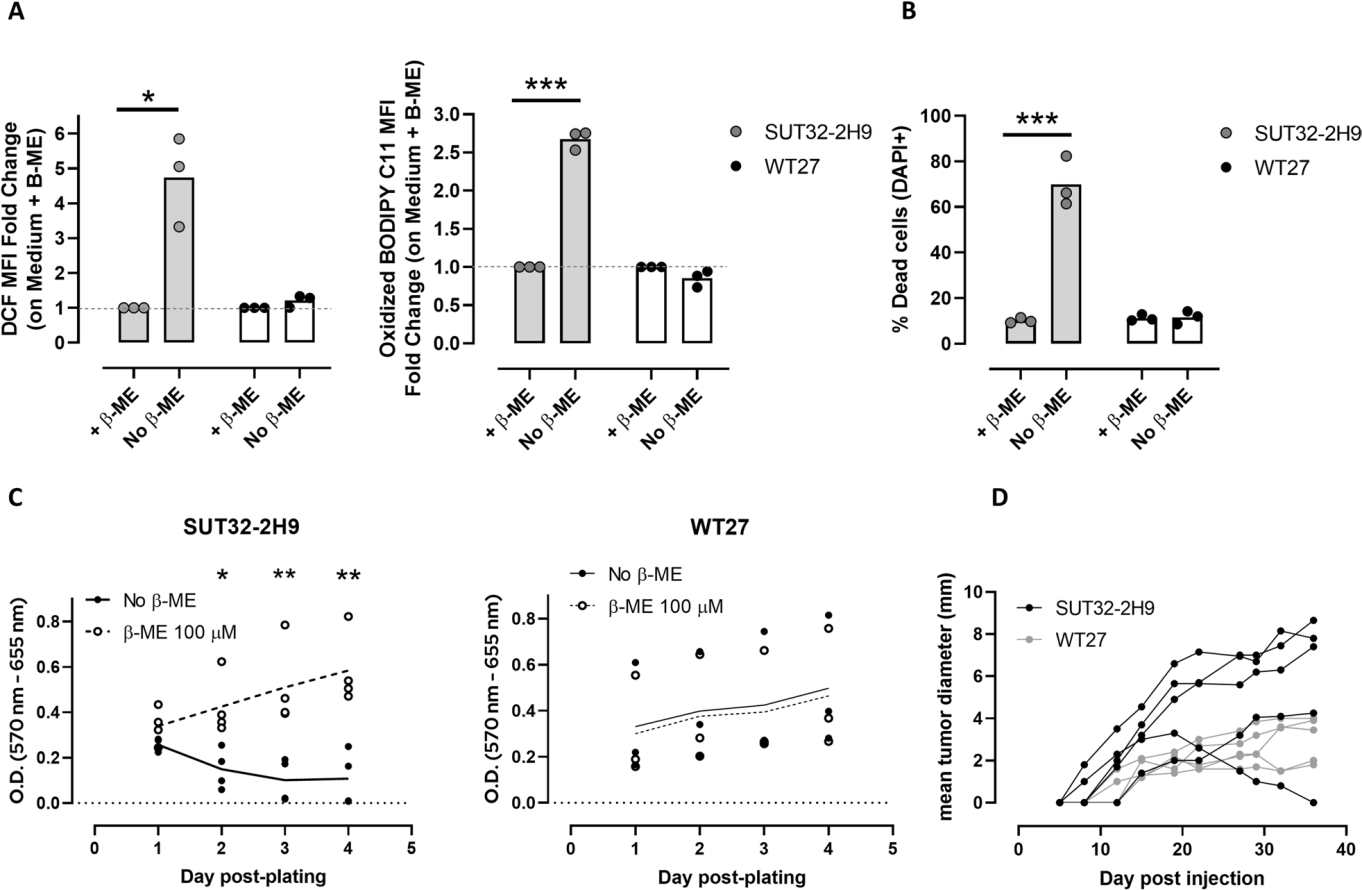
Generation and characterization of cell lines from BALB-neuT/xCT^wt^ and BALB-neuT/xCT^null^ tumors. **A** Fold change of the mean fluorescent intensity (MFI) of DCF signal or Oxydized Bodipy C11 signal 72 h after removal or not of β-ME from the growth medium, assessed through FACS analysis. **B** Percentage of dead (DAPI+) cells 72 h after removal of β-ME from the growth medium. **C** Proliferation curves, assessed by MTT assay, of SUT32-2H9 cells or WT27 cells in the presence or absence of β-ME. **D** Growth curves of 1 x 10^6^ SUT32-2H9 or WT27 cells injected subcutaneously in BALB/c mice. Number of replicates: each dot depicts a mouse (*n* = 5 per group in panel D) or an independent biological replicate. Statistical analysis: ratio paired t test (panel A) or unpaired t test (panels B and C). * *p*<0.05; ** *p*<0.01; *** *p*<0.001; Where not indicated, *p* value is not significant. Histograms (panels A, B) or lines (panel C) represent mean values. Error bars are not shown as n < 5

To assess whether xCT depletion may have discrepant effects on cancer cells in vitro versus in vivo as for immune cells, we generated a cancer cell line from a BALB-neuT/xCT^null^ tumor (SUT32-2H9, Supplementary Fig. S3A). When compared to cancer cells derived from BALB-neuT/xCT^wt^ mice (WT27), SUT32-2H9 cells showed increased susceptibility to oxidative stress and lipid peroxidation (Fig. 3A), as well as survival (Fig. 3B) and proliferation (Fig. 3C) defects that were rescued by the addition of β-ME, indicating that oxidative stress is at the basis of this phenotype. Re-expression of xCT by a lentiviral vector completely rescued the proliferative defect in SUT32-2H9 cells (Supplementary Fig. S3B), thus strengthening the hypothesis that xCT is required for cancer cell proliferation in vitro. The use of Human Plasma-Like Medium (HPLM, which more closely recapitulates the nutrients and metabolites available in plasma [34]) did not alter susceptibility of xCT^null^ cells to cell death (Supplementary Fig. S3C), indicating that it is dependent on factors related to in vitro culturing and not on the medium composition. Indeed, challenging BALB/c-xCT^wt^ mice with SUT32-2H9 cells resulted in 100% tumor take, and most tumors grew progressively as for WT27 cells (Fig. 3D), suggesting the presence of compensatory mechanisms in vivo. On the other hand, re-expression of xCT in SUT32-2H9 cells did not provide an advantage in tumor take and growth (Supplementary Fig. S3D).

**Fig. 4:**
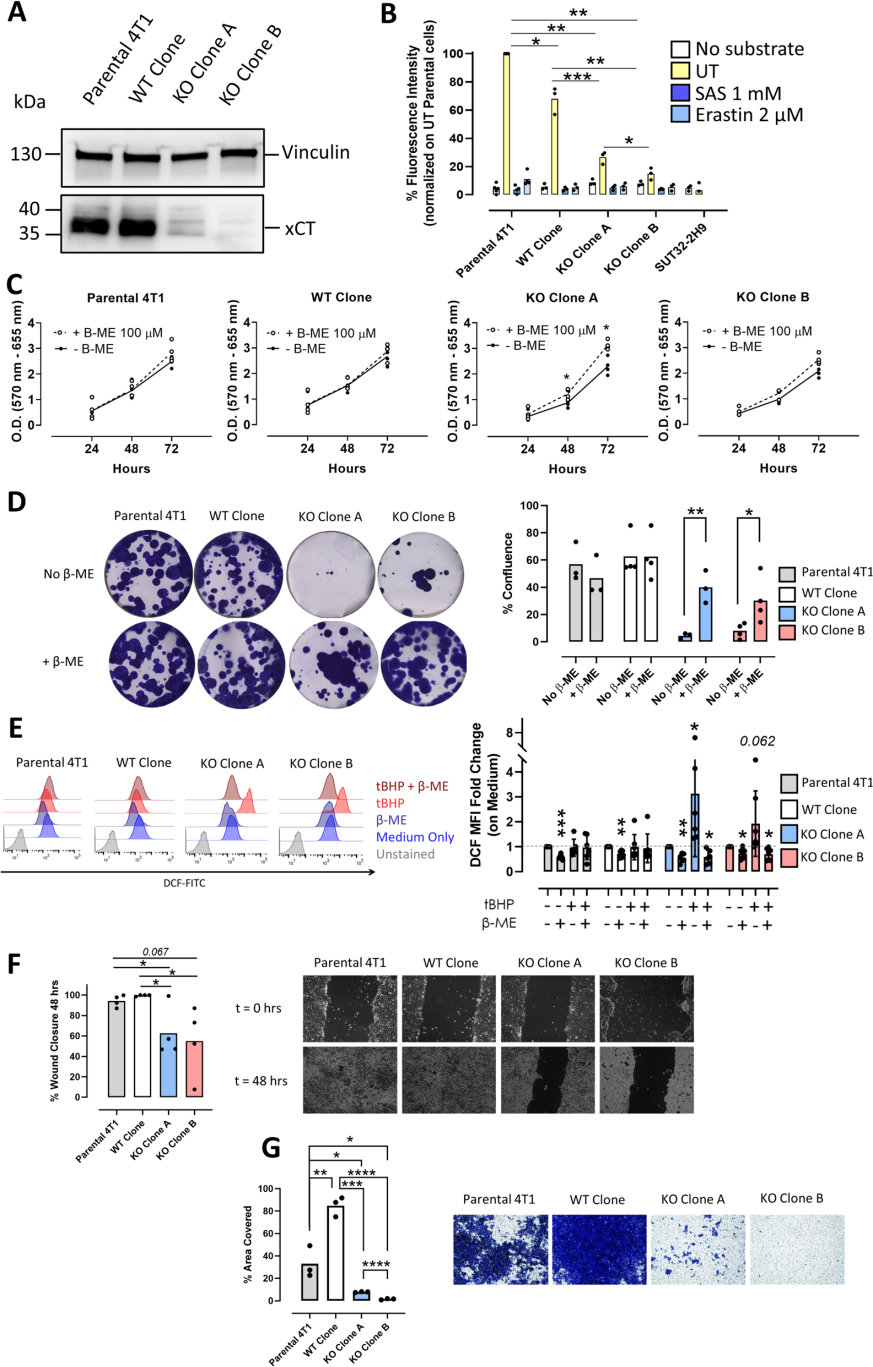
Generation and characterization of 4T1 xCT^KO^ cell clones. **A** Western blot analysis of xCT expression, using vinculin as loading control. **B** Selenocystine uptake assay (used as a surrogate of cystine uptake), expressed as % fluorescence emitted as compared to parental 4T1 cells. Cells incubated with SAS or Erastin are used as control. **C** Cell proliferation curves of parental 4T1 cells, WT and KO clones, assessed by MTT assay. **D** Left: Colony-forming efficiency assay. Right: Percentage of plate surface occupied by colonies. **E** Left: FACS analysis of ROS content following 4 h incubation with 100 µM of tBHP, indicated by 2’,7’-Dichlorofluorescin diacetate (DCF-DA) fluorescent signal. Right: DCF fluorescent signal represented as fold change of DCF MFI, normalized on cells incubated in growth medium alone. **F** Right: Representative images of migrating cells in a wound-healing assay at 0 and 48 h post scratching. Left: Percentage of wound closure (of initial wound area) at 48 h. **G** Left: Percentage of transwell area covered by migrated cells. Right: Representative images of migrating cells in a transwell migration assay. Number of replicates: each dot represents an independent biological replicate, which is the result of at least two technical replicates, except for experiments of flow cytometry, where only a technical replicate for biological sample was performed. Statistical analysis: unpaired t test (panels C-F, G) or ratio paired t test (panels B, E). *p<0.05; ** *p*<0.01; *** *p*<0.001; **** *p*<0.0001. Where not indicated, *p* value is not significant, except in panel B, where dots and lines depicting comparisons with negative controls are omitted for a better visualization. Lines (panel C only) and histograms represent mean values. Error bars are shown only when n > 5, and represent SD

We then used 4T1 cells as a second, well-characterized, mouse mammary cancer model for the validation of our previous findings. We generated xCT^KO^ cells using CRISPR/Cas9 technology and selected clones A and B, which exhibited successful *Slc7a11* gene disruption, as shown by the lack of xCT protein (Fig. 4A) and the strong reduction of xCT mRNA levels (Supplementary Fig. S4A), compared to parental 4T1 cells and to a xCT^wt^ clone obtained using a non-targeting CRISPR/Cas9 vector. As expected, cystine uptake was strongly reduced in xCT^KO^ clones compared to 4T1 xCT^wt^ cells, either parental or cloned (Fig. 4B). The xCT inhibitors SAS and Erastin were used as controls.

Surprisingly and contrary to what was found by us with SUT32-2H9 cells and by others with tumor cell lines of other histotypes [18], the absence of xCT did not significantly impact short-term proliferation, except for clone A, which exhibited a moderate reduction in proliferation (Fig. 4C). Nevertheless, in the absence of β-ME, xCT^KO^ clones displayed impaired ability to generate single-cell colonies when seeded at a very low density (Fig. 4D).

xCT^KO^ clones showed an increased level of ROS (Fig. 4E and Supplementary Fig. S4B), a trend of increased lipid peroxidation (Supplementary Fig. S4C, D), and extensive cell death (Supplementary Fig. S4E) 4 h after the administration of the oxidative compound tert-Butyl hydroperoxide (tBHP), as compared to 4T1 parental cells and the xCT^wt^ clone. β-ME supplementation of the culture medium completely rescued xCT^KO^ cell sensitivity to oxidative stress.

The non-lethal phenotype of xCT^KO^ 4T1 cells allowed us to extend our study on xCT function to other malignant features, as 4T1 cells are a renowned model for metastatic breast cancer, with a marked migratory phenotype [35]. According to the link between xCT function and cancer cell migration [20], xCT deficiency impaired the migratory ability of 4T1 cells in vitro (Fig. 4F, G). A pool of 10 additional xCT^KO^ clones (Supplementary Fig. S4F) showed the same migratory defects (Supplementary Fig. S4G). Conversely, mild overexpression of xCT in 4T1 cells (Supplementary Fig. S4H) further increased their migratory ability (Supplementary Fig. S4I) without affecting their proliferation rate (Supplementary Fig. S4J).

Overall, these data indicate that xCT is required for the long-term maintenance of cancer cells in vitro and its depletion causes excessive oxidative stress in the presence of oxidative compounds. In addition, xCT promotes the in vitro migratory ability of cancer cells.

### xCT depletion in cancer cells impairs lung metastasis formation and alters the metastatic niche

We then analyzed the role of xCT in 4T1 cells in vivo, in terms of tumor take and metastasization. As compared to the xCT^wt^ clone and xCT^KO^ Clone B, xCT^KO^ Clone A exhibited a reduction in both tumor growth rate and tumor weight (Supplementary Fig. S5A, B). However, similar to the xCT^wt^ clone, both xCT^KO^ clones generated primary tumors when injected subcutaneously; however, they were less able to metastasize from the primary site as compared to the xCT^wt^ clone (Supplementary Fig. S5C).

**Fig. 5:**
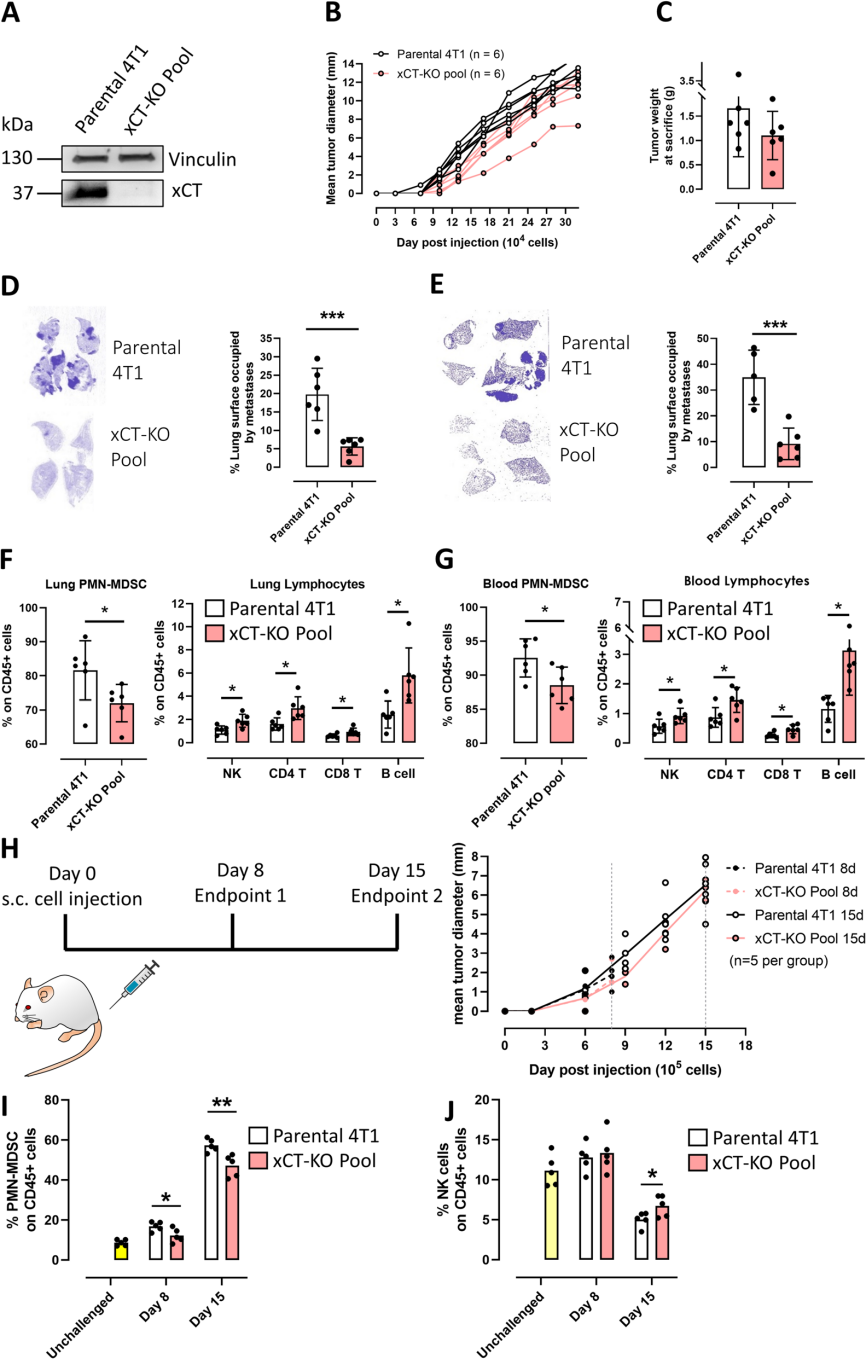
xCT depletion in cancer cells impairs metastasis formation and alters the metastatic niche*. ***A** Western blot analysis of xCT expression in WT 4T1 cells and the pool of 10 xCT^KO^ clones (xCT-KO Pool). Vinculin is used as loading control. **B** Growth curves of tumors deriving from 10.000 4T1 (WT or KO pool) injected subcutaneously in BALB/c mice. **C** Tumor weight at sacrifice. **D** Left: representative slices of FFPE lungs from mice described in panel B, stained with H&E. Right: percentage of slice area occupied by metastases. **E** Left: representative slices of FFPE lungs (following i.v. injection of 10.000 4T1, either WT or KO pool) stained with H&E. Right: percentage of slice area occupied by metastases. Percentage of selected immune cell populations over total leukocytes (CD45+) **F** infiltrating the lungs or **G** isolated from peripheral blood of tumor-bearing mice described in panel B. **H** Left: experimental protocol used to assess alterations in the immune pre-metastatic niche. Right: Mean tumor growth curves of mice are shown for a better visualization. Percentage of **I** PMN-MDSC or **J** NK over total leukocytes (CD45+) infiltrating the lungs of tumor-bearing mice (described in panel H) at different stages of tumor growth and of healthy, unchallenged mice. Number of replicates: number of mice is reported in panels B and H; each dot represents a mouse. For flow cytometry data, each dot depicting a mouse is the result of a single technical replicate. Statistical analysis: unpaired t test. * *p*<0.05; ** *p*<0.01; *** *p*<0.001. Where not indicated, *p* value is not significant. Lines (panel H only) and histograms represent mean values. Error bars are shown only when n > 5, and represent SD

Because of the observed differences between clones A and B, and the very heterogeneous phenotype of 4T1 parental cells [36], we used the 10 additional xCT^KO^ clones (Supplementary Fig. S3D) pooled together to mimic 4T1 heterogeneity and mitigate any clonal difference (Fig. 5A). When injected subcutaneously, parental 4T1 and the xCT^KO^ pool generated tumors with comparable growth rates (Fig. 5B) and weight at sacrifice (Fig. 5C). In tumor-bearing mice, spleen weight (Supplementary Fig. S5D) and overall level of tumor-infiltrating immune cells were also similar (Supplementary Fig. S5E). Nevertheless, xCT depletion strongly reduced the formation of spontaneous lung metastases from the primary subcutaneous site (Fig. 5D), confirming the observations reported with the xCT^KO^ clones A and B. In vivo, slower-growing clones within the pool might be concealed by faster-growing ones, thus leading to a reduced pool heterogeneity by the end of the experiment. To assess if and how many clones relied on xCT for in vivo growth, we subcutaneously challenged mice with 10.000 cells from each individual clone, besides parental 4T1 cells and the xCT^KO^ pool. Parental 4T1 cells and the xCT^KO^ pool displayed superimposable growth curves (Supplementary Fig. S5F). Focusing on the single clones, some of them displayed growth profiles similar to the parental 4T1 cells (1C1, 1E10, 2A11), some others exhibited slow growth (3H2, 1E1, 3A5, 1B5), while another group demonstrated incomplete tumor take and slow growth (1H8, 3G5, 1F5). This suggests that, over time, clones with rapid growth within the pool could potentially outpace their slower-growing counterparts. Nevertheless, each group exhibited at least one tumor with observable growth (Supplementary Fig. S5G), confirming that xCT is dispensable for primary tumor initiation. When analyzing spontaneous lung metastases, consistent trends emerged. Despite certain clones’ ability to establish primary tumors, 8 out of 10 clones were unable to induce significant metastatic occurrences, even with primary tumor sizes comparable to those originating from parental 4T1 cells. Also in this experiment, xCT^KO^ pool was unable to form a substantial number of metastases, contrarily to the parental 4T1 cells (Supplementary Fig. S5H). Similarly, intravenously injected xCT^KO^ cells had an impaired ability to form lesions in the lungs (Fig. 5E). Re-expression of xCT through a lentiviral vector partially rescued the metastatic ability of xCT^KO^ cells (Supplementary Fig. S5I).

Previous research suggested the ability of xCT to modulate the extracellular environment through cystine uptake or glutamate secretion [16, 19], as well as through the autocrine/paracrine activation of machineries involved in the secretion of cytokines and EV [20–22]. Thus, we analyzed the immune subpopulations in the blood and lungs of mice challenged subcutaneously with 4T1 cells to determine whether xCT may contribute to the formation of the metastatic niche through the recruitment and polarization of immune cells. BALB/c-xCT^wt^ mice challenged with the xCT^KO^ pool showed an increase in the proportion of CD4^+^ T, CD8^+^ T, B, and NK cells and a reduction in neutrophils/PMN-MDSC in both the blood and lungs (Fig. 5F, G and Supplementary Fig. S6A, B). In the primary tumor, xCT depletion in cancer cells resulted in an increase of CD8^+^ T lymphocyte infiltration (Supplementary Fig. S6C).

To assess whether these alterations in immune cell infiltration in the lungs are a consequence of metastasis outgrowth or precede metastasis formation, we analyzed the immune infiltrate in the lungs of cancer-bearing mice 8- and 15-days post-challenge (Fig. 5H) before overt lung metastasis formation. We observed again a decrease in the percentage of PMN-MDSC (Fig. 5I) and an increase in NK cells (Fig. 5J) in mice challenged subcutaneously with xCT^KO^ compared to parental 4T1 cells, indicating that xCT^KO^ cells are defective in recruiting an immune-suppressive metastatic niche.

To identify the possible mediators of this (pre)metastatic niche alteration, we tested cytokine release. The xCT^KO^ clones showed reduced in vitro release of VEGF and GM-CSF, while TGF-β and G-CSF decreased only in KO Clone A and thus cannot be considered xCT-dependent (Supplementary Fig. S6D). In vivo, we observed a comparable increase in G-CSF plasma concentration in both 4T1 and 4T1 xCT^KO^-bearing mice (Supplementary Fig. S6E, F), and the analysis of other plasma cytokines did not show differences between the two groups (Supplementary Fig. S6F). We were not able to detect GM-CSF and VEGF in the plasma.

Collectively, our data demonstrate that xCT depletion reduces the metastatic ability of mammary cancer cells, whereas primary tumor formation and progression are not affected in our experimental context.

## Discussion

Although previous reports have indicated that xCT depletion in tumor cells leads to impaired tumor growth in vivo [18], our data obtained in mammary cancer-prone BALB-neuT mice show that congenital, total body xCT deficiency does not affect the time of onset or the multiplicity of tumors, but reduces the incidence of lung metastases. To date, only two studies have evaluated the consequences of xCT deficiency in tumor progression in cancer-prone mice, both using the KPC pancreatic tumor model. In the first study [37], tamoxifen-induced whole-body *Slc7a11* KO after tumor appearance led to increased survival. In the second study [38], congenital epithelial-specific *Slc7a11* KO did not alter the incidence, onset, and progression of pancreatic cancer. These differences suggest a pivotal role for xCT in the tumor stroma during tumor progression, or, alternatively, that the timing of xCT deletion may be crucial for the tumor outcome. However, the striking impairment in proliferation, increased ROS content, and lipid peroxidation observed in vitro under basal conditions in tumor cells derived from BALB-neuT/xCT^null^ mice, and the finding that the addition of β-ME or re-expression of *Slc7a11* is sufficient for the reversion of these phenotypes reveals indeed essential functions of xCT in the biology of these epithelial tumor cells under specific conditions. Thus, it seems likely that xCT deficiency obtained prior to the onset of neoplastic transformation promotes the occurrence of compensatory mechanisms that allow cells to bypass the defective cystine/glutamate exchange in vivo. Although the nature of such compensatory mechanisms remains to be identified, the different environmental conditions of cultured cells, such as supraphysiological oxygen [39], likely represent a challenging environment that may increase the oxidative stress that cells must face compared to an in vivo environment. Moreover, plasma and culture media contain different concentrations of cystine, cysteine, and GSH. Whereas cysteine is absent in the culture medium, it is present in the plasma [40], and can be imported by cells via alternative transporters (e.g., ASCT1, ASCT2 [41], LAT1 [5]), thus circumventing the requirement of system xc^−^. Hence, it is possible that cysteine in plasma could provide a sufficient support in vivo to immune system functionality on one hand, and to tumor initiation and growth on the other hand.

In sharp contrast to the results obtained with tumor cells derived from xCT^null^ mice, we did not observe significant impairments in xCT^KO^ 4T1 proliferation in vitro under standard confluence culture conditions. Increased oxidative stress in xCT^KO^ cells, but not in xCT^wt^ 4T1 cells, was observed only upon the administration of tBHP, and this was rescued by the addition of β-ME. This indicates that xCT^KO^ 4T1 cells have indeed a latent imbalance in redox homeostasis, which becomes overt only under stress conditions. 4T1 cells are thus endowed with compensatory mechanisms that allow them to survive and proliferate under normal culture conditions, but that are not sufficient to overcome further increases in oxidative stress. These processes may include the *de novo* synthesis of cysteine via the transsulfuration pathway [42] or the expression of other cystine transporters. These may include the heterodimers SLC3A1/SLC7A9 and SLC3A1/SLC7A13, and the excitatory amino acid transporters (EEAT) [43]. However, the relevance of such processes in tumor biology is currently unclear, and the specific mechanisms that allow 4T1 cells to bypass xCT deficiency under basal growth conditions in vitro still need to be identified.

The 4T1 model is highly metastasis-prone and thus represents a better experimental context to assess the contribution of xCT to the metastatic process than the poorly metastatic BALB-neuT model [29], which nevertheless displays a trend of reduced incidence of metastasis within the ethical endpoint when xCT is absent. Importantly, xCT depletion in 4T1 cells resulted in reduced migration in vitro and in a strong impairment of cell metastatic ability in vivo, which is consistent with previous reports [44, 45]. Notably, xCT^KO^ 4T1 cells showed a dramatic impairment in their clonogenic ability in vitro, which may play a key role in the conversion of seeded cancer cells into overt metastases in vivo. As shown recently, ferroptosis limits the survival of cancer cells in the bloodstream owing to the high free iron concentration [46], thus providing a rational explanation for the drastic impairment of the metastatic ability of xCT^KO^ cells, in spite of the lack of significant differences between primary tumors generated by them and those of xCT^wt^ cell counterparts. Moreover, xCT-dependent glutamate secretion by breast cancer cells was found to induce autocrine/paracrine activation of metabotropic glutamate receptor (GRM)3/Rab27a-dependent membrane trafficking [20]. This leads to the relocation and secretion of invasion-promoting proteins that allow breast cancer cells to metastasize [20]. Thus, the overall impairment of the metastatic ability of xCT^KO^ 4T1 cells may result from both an increased propensity to undergo ferroptosis in the bloodstream owing to a reduced ROS-buffering capacity, and an impaired cell migration secondary to a blunted glutamate secretion.

In addition to this cell-autonomous mechanism, Rab27a activity in 4T1 cells mediates secretion of EV and cytokines that recruit tumor-promoting neutrophils [47]. Interestingly, xCT depletion in 4T1 tumor cells resulted in a significant increase in CD4^+^ T, CD8^+^ T, B, and NK cells, which are important players in the antitumor immune response [48], and in fewer PMN-MDSC, in the lungs. This was not obvious in xCT^wt^ and xCT^null^ BALB-neuT mice [29], where the presence of PMN-MDSC in the lungs was much lower than that induced in 4T1 tumor-bearing mice, in line with BALB-neuT reduced propensity to develop lung metastases. PMN-MDSC are reportedly involved in the promotion of metastasis by participating in the formation of pre-metastatic niches [49]; their expansion is induced by different stimuli including G-CSF and GM-CSF [50]. We indeed observed an increase in the amount of G-CSF in the plasma of tumor-bearing compared to that of healthy mice, but not in mice challenged with xCT^wt^ 4T1 cells versus xCT^KO^ cells. Besides cytokines, other in vivo mechanisms may thus be responsible for differential MDSC expansion, such as EV released in an xCT-dependent manner [21]. EV mediate communication between primary tumors and distant organs, and it has already been reported that breast cancer-derived EV stimulate MDSC expansion [51], and that xCT is involved in EV release from transformed cells [20–22]. Further experiments are warranted to elucidate the possible mechanisms underlying the observed effects on the metastatic ability.

Given that high xCT expression in tumors is associated with a poor prognosis in oncological patients [9, 12], its targeting has been investigated by us and others [6, 14, 52] as a therapeutic strategy. xCT is also expressed in several types of activated immune cells [5, 15, 17]. Despite this, our results demonstrate that the proportions of different immune cell populations were not altered in healthy xCT^null^ mice. In addition, cellular and humoral immune responses against non-self-antigens were preserved in the vaccinated xCT^null^ mice and in mice treated with the xCT inhibitor SAS. Accordingly, systemic xCT deficiency does not alter the composition of the immune infiltrate in the primary tumor in vivo; thus, xCT is dispensable for immune system function. This is in line with a previous report by Arensman et al. [18], although they focused exclusively on the T-cell response. These data indicate that xCT targeting would be effective in tumor cells, while sparing cells of the immune system. Although pharmacological or immune-mediated inhibition of a protein does not necessarily recapitulate the disruption of its coding gene, our results corroborate our previous observations that anti-xCT vaccination efficiently impairs tumor metastasization while preserving antitumor immunity [13, 14].

## Conclusions

Our data demonstrate that xCT deficiency does not hinder the initiation and progression of mammary cancer. However, it does sensitize cancer cells to oxidative stress, and most notably, inhibits the formation of metastases, which are the primary cause of cancer-related deaths. Moreover, our findings suggest a non-cell-autonomous role of xCT in breast cancer malignancy, specifically a connection between xCT expression and the composition of the (pre)metastatic niche. Further research focused on unraveling the underlying mechanisms of this association may broaden the scope of the xCT-targeting therapeutic strategies currently under investigation. These strategies encompass the clinically approved drug SAS, as well as other molecules currently in the pre-clinical stage, such as anti-xCT vaccines [14]. Implementing these therapeutic approaches would not only impair the intrinsic malignant behavior of cancer cells but also counteract the development of metastasis-supporting niches.

### Supplementary Information

**Additional file 1.** Supplementary figures S1, S2, S3, S4, S5, S6.

**Additional file 2.** Supplementary Methods; Key resources table; SI References.

**Additional file 3.** Uncropped western blots.

## Data Availability

All unique reagents, cell and mouse lines generated in this study will be made available on request with a completed materials transfer agreement and with reasonable compensation by requestor for their processing and shipping. We may require a payment if there is potential for commercial application. All data reported in this paper and any additional information required to reanalyze the data reported in this paper will be shared by the corresponding author, Federica Cavallo (federica.cavallo@unito.it) upon request. This paper does not report original code. A complete table of reagents and resources used in this study is reported in the Additional file 2.

## Abbreviations

ASCT1/ASCT2: Amino Acid Transporter System 1/2
ATCC: American Type Culture Collection
BM: Bone Marrow
Cas9: CRISPR-associated protein 9
CM: Conditioned medium
CMV: Cytomegalovirus
CRISPR/Cas9: Clustered Regularly Interspaced Short Palindromic Repeats/CRISPR-associated protein 9
CFSE: Carboxyfluorescein succinimidyl ester
DMEM-F12: Dulbecco’s Modified Eagle’s Medium/Nutrient Mixture F-12
ELISA: Enzyme-Linked Immunosorbent Assay
ERBB2: Erb-B2 Receptor Tyrosine Kinase 2 (also known as HER2)
EV: Extracellular Vesicles
FBS: Fetal Bovine Serum
G-CSF: Granulocyte Colony-Stimulating Factor
GM-CSF: Granulocyte-Macrophage Colony-Stimulating Factor
GRM3: Metabotropic Glutamate Receptor 3
GSH: Glutathione
HPLM: Human Plasma-Like Medium
HRP: Horseradish Peroxidase
i.v.: Intravenous
IgG: Immunoglobulin G
KPC: Kras^G12D/+; Trp53^R172H/+; Pdx1-Cre
LAT1: Large Neutral Amino Acid Transporter 1
MDSC: Myeloid-Derived Suppressor Cells
MHC-I: Major Histocompatibility Complex class I
NK: Natural Killer
P/S: Penicillin/Streptomycin
PMN-MDSC: Polymorphonuclear Myeloid-Derived Suppressor Cells
PVDF: Polyvinylidene Fluoride
Rab27a: Ras-Related Protein Rab-27 A
RHuT: Rat/Human chimeric form of ERBB2
RIPA: Radioimmunoprecipitation Assay
ROS: Reactive Oxygen Species
SAS: Sulfasalazine
s.c.: Subcutaneous
SLC7A11: Solute Carrier Family 7 member 11
tBHP: tert-Butyl hydroperoxide
TGF-β: Transforming Growth Factor-beta
Treg: Regulatory T cells
VEGF: Vascular Endothelial Growth Factor
xCT: Cystine/Glutamate Transporter (also known as SLC7A11)
β-ME: Beta-Mercaptoethanol
