# Supplementary material for: Cystine/glutamate antiporter xCT deficiency reduces metastasis without impairing immune system function in breast cancer mouse models
Source: J Exp Clin Cancer Res. 2023 Sep 29;42:254. doi: 10.1186/s13046-023-02830-x (PMC10540318; doi:10.1186/s13046-023-02830-x)
Supplement: Supplementary file 1 — Additional file 1. Supplementary figures S1, S2, S3, S4, S5, S6. [file 13046_2023_2830_MOESM1_ESM.docx]

**Supplementary Figures from**

**Cystine/glutamate antiporter xCT deficiency reduces metastasis without impairing immune system function in breast cancer mouse models**

Roberto Ruiu^1^, Chiara Cossu^1^, Antonella Iacoviello^1^, Laura Conti^1^, Elisabetta Bolli^1^, Luca Ponzone^2^, Jolanda Magri^1^, Alekya Rumandla^1^, Enzo Calautti^2^, Federica Cavallo^1^

*^1^Laboratory of Oncoimmunology, Molecular Biotechnology Center “Guido Tarone”, Department of Molecular Biotechnology and Health Sciences, University of Turin, Turin, Italy*

*^2^Laboratory of Epithelial Stem Cell Biology and Signaling, Molecular Biotechnology Center “Guido Tarone”, Department of Molecular Biotechnology and Health Sciences, University of Turin, Turin, Italy*

Corresponding author

Federica Cavallo, Molecular Biotechnology Center “Guido Tarone”, Via Nizza, 52, Turin 10126, Italy. Phone: +39 011 670 6457; Fax: +39 011 236 6457; E-mail: [federica.cavallo@unito.it](mailto:federica.cavallo@unito.it)


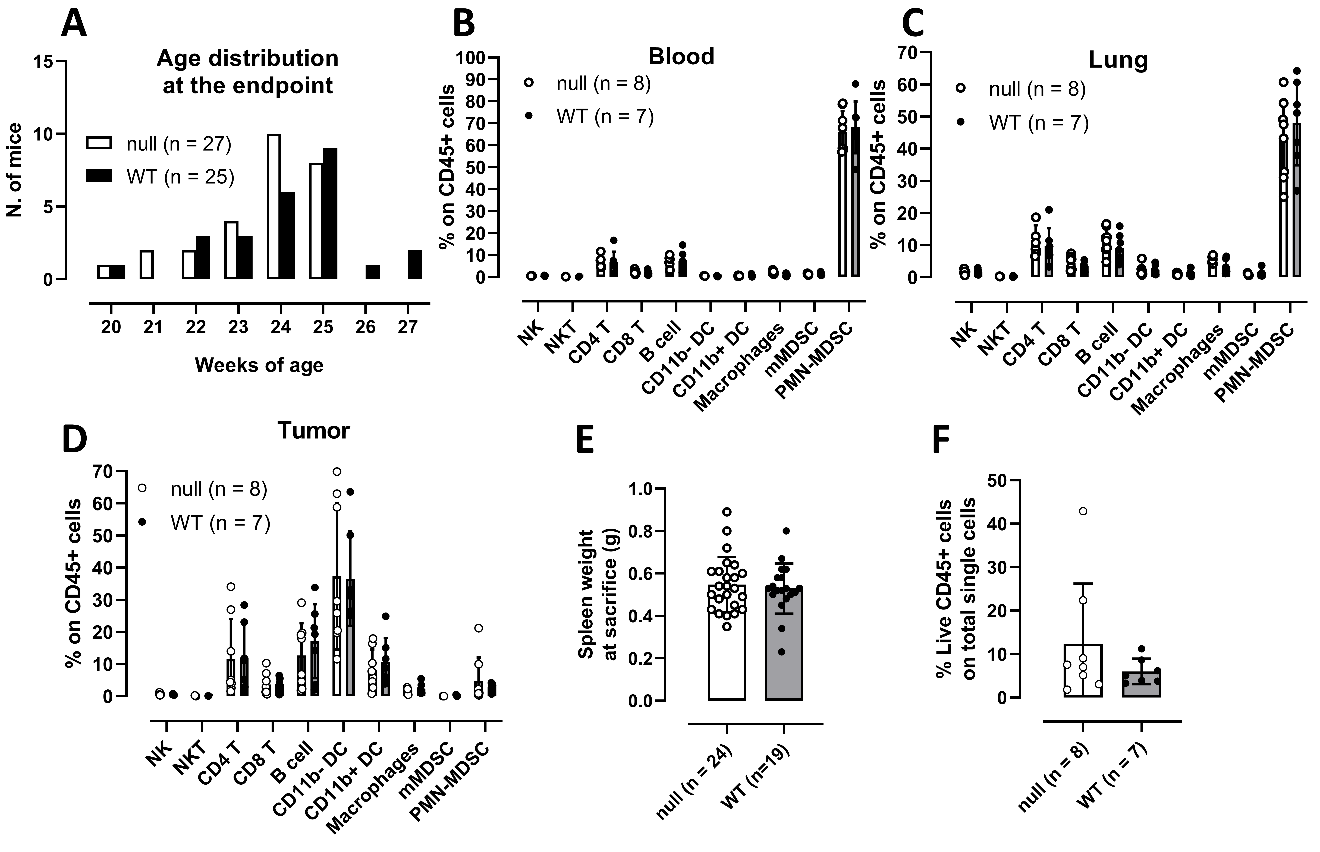


Fig. S1. Immune cell populations and immune response to tumors in xCT^wt^ or xCT^null^ BALB-neuT mice.

**A** Distribution of mice according to age (expressed in weeks) at sacrifice for ethical reasons. Leukocyte populations proportions on total leukocytes (CD45+) isolated from **B** blood, **C** lung or **D** tumor of BALB-neuT/xCT^wt^ (WT) and BALB-neuT/xCT^null^ (null) mice, as assessed by flow cytometry. **E** Spleen weight at the sacrifice of BALB-neuT/xCT^wt^ (WT) and BALB-neuT/xCT^null^ (null) mice. **F** Infiltration of leukocytes within tumors isolated from BALB-neuT/xCT^wt^ (WT) and BALB-neuT/xCT^null^ (null) mice, as assessed by flow cytometry. Optical Densities (O.D.) of dissolved MTT crystals are used as a readout of live cells. Number of replicates: number of mice studied is reported in the panel legend, and each dot represents a mouse. For flow cytometry data, each dot depicting a mouse is the result of a single technical replicate. Statistical analysis: unpaired t test (panels B, C, D, E, F): if not indicated, *p* value is not significant. Histograms represent mean values. Error bars are shown only when n > 5, and represent SD.

**
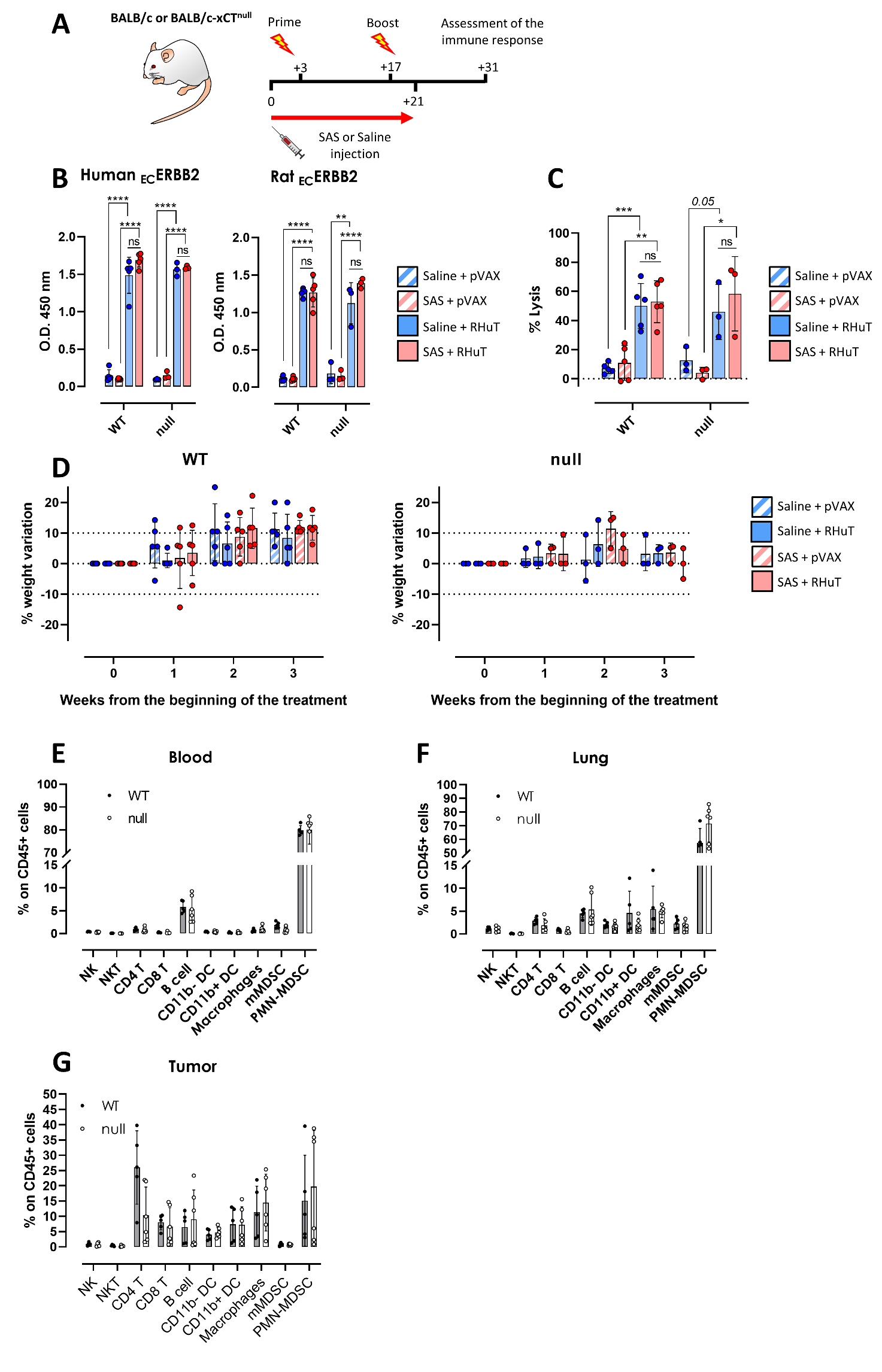
**

Fig. S2. Effect of xCT genetic ablation or pharmacological inhibition on adaptive immune response and immune cell proportions.

**A** Treatment schedule of xCT^wt^ (WT) and xCT^null^ (null) BALB/c mice with the empty pVAX1 or the RHuT plasmids, in combination with SAS or saline. **B** ELISA assay of sera from treated mice, recognizing Human or Rat ERBB2 extracellular (EC) portion. Intensity of colorimetric signal is represented by optical density (O.D.) measured at 450 nm. **C** Percentage of lysis of splenocytes stained with CFSE, pulsed with the target antigen immunodominant peptide, and injected in mice immunized with pVAX1 or RHuT and treated with SAS or saline, as assessed by flow cytometry. The percentage of lysis is normalized on the proportion of antigen-pulsed and non-pulsed splenocytes injected and then extracted from untreated mice, not shown in the graph. **D** Percentage of weekly weight variation in WT (left) or null (right) mice, normalized for each mouse on the weight of the same mouse in the week when treatment started. Percentage of immune cell populations in **E** blood, **F** lung, and **G** tumor from mice described in Fig.2N. Number of replicates: Each dot represents a mouse and is the result of a single technical replicate for flow cytometry and of a technical duplicate for the ELISA. In experiments described in panels A-D, 5 WT and 3 null BALB/c mice per group were used. In panels E-G, the number of WT mice used is 5 instead of 6 since one mouse died just before the endpoint. Statistical analysis: unpaired t test. In panels A-C, comparisons between different treatments and different genotypes were performed. In panel D, comparisons between different treatments were performed per single time points. ** *p*<0.01; *** *p*<0.001; **** *p*<0.0001; ns = non-significant. In panels D-G and in genotype comparisons within panels B and C, when the p-value is not significant, it is not denoted as "ns" This omission is made to enhance the clarity of visualization, even though the comparison has been executed. Histograms represent mean values. Error bars represent SD.


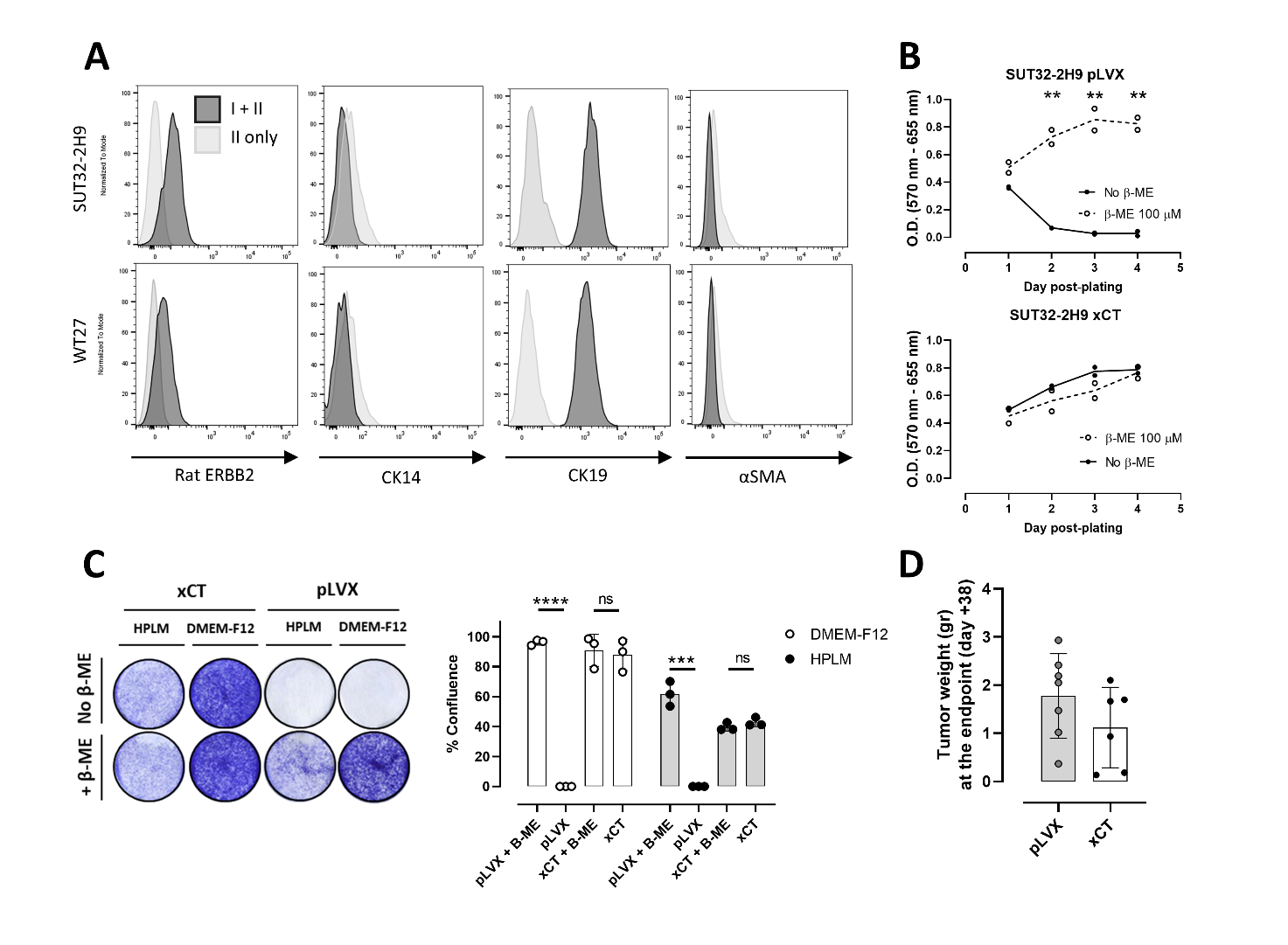


Fig. S3. Generation and characterization of SUT32-2H9 and WT27 cells.

**A** Flow cytometry analysis of rat ERBB2, CK14, CK19, α-SMA expression on neuT/xCT^null^ (SUT32-2H9) or neuT/xCT^wt^ (WT27) cells. Light gray: isotype-specific fluorochrome-conjugated antibody only; dark gray: antigen-specific primary antibody + isotype-specific fluorochrome-conjugated antibody. **B** Proliferation curves, assessed by MTT assay, of SUT32-2H9 cells transduced with an empty lentiviral vector (pLVX) or an xCT-expressing lentiviral vector (xCT), in the presence or absence of β-ME in the growth medium. **C** Left panel: Crystal violet staining of SUT32-2H9 cells transduced with an empty (pLVX) or xCT-expressing (xCT) lentiviral vector, cultured for 72 hrs in DMEM-F12 supplemented with 20% FBS or in HPLM supplemented with 20% dialyzed FBS, with or without 100 µM β-ME; Right panel: quantification of cell confluence depicted in the left panel. **D** Tumor weight at the endpoint, from female BALB/c mice challenged with a s.c. cell injection of 100.000 SUT32-2H9 pLVX or SUT32-2H9 xCT cells. Number of replicates: each dot (Panels B and C) represents an independent biological replicate, each resulting from at least a technical duplicate. In panel D, each dot represents a distinct mouse, histograms represent mean values and error bars represent SD. Statistical analysis: unpaired t test. ** *p*<0.01; *** *p*<0.001; **** *p*<0.0001; ns: not significant. Where not indicated, *p* value is not significant.


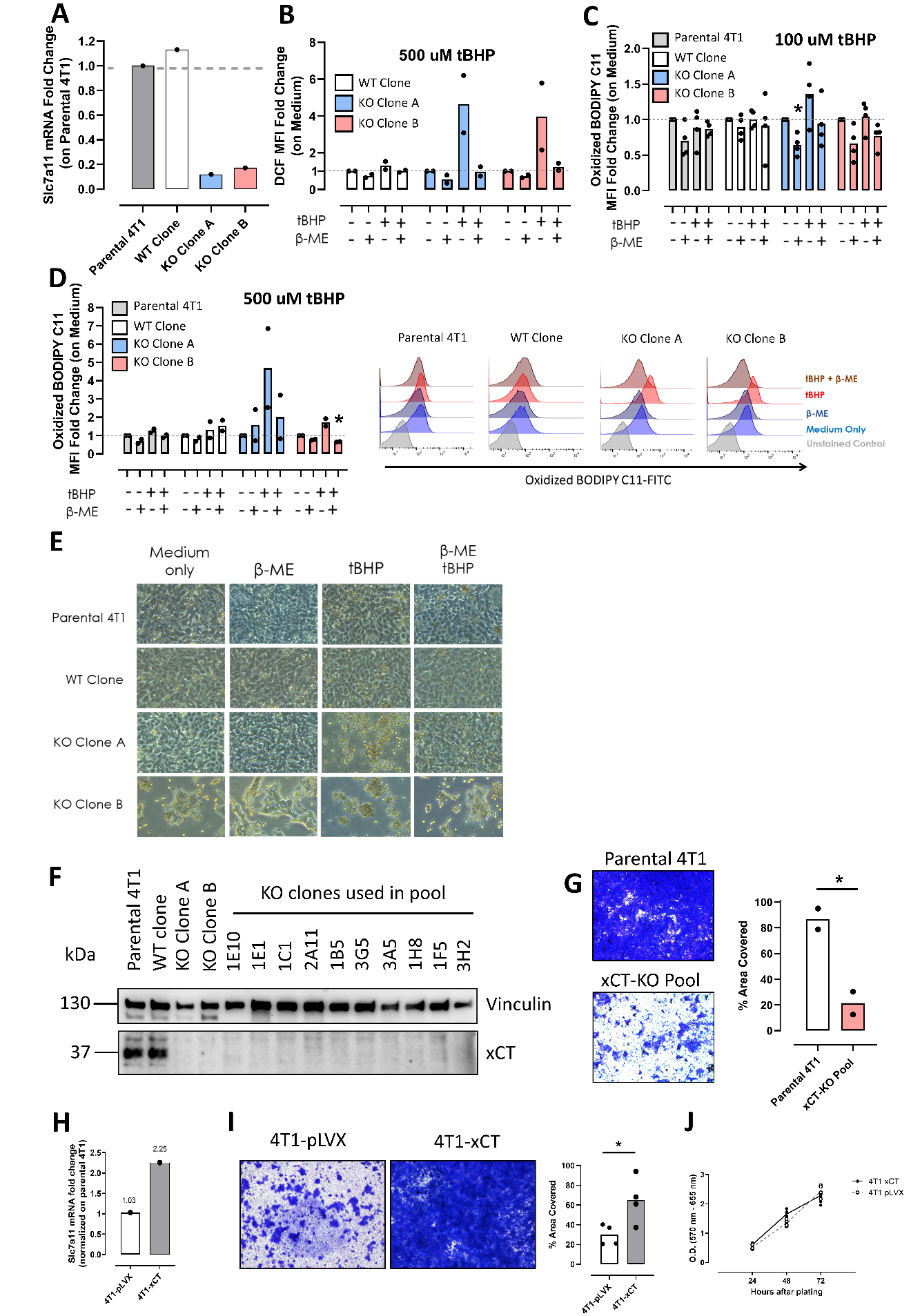


Fig. S4. Generation and characterization of 4T1 xCT^KO^ cell clones.

**A** *Slc7a11* transcript expression levels in WT and KO clones, represented as fold change compared to *Slc7a11* transcript levels in parental 4T1 cells. **B** Analysis of reactive oxygen species content following 4 hrs incubation with 500 µM of tBHP, indicated by DCF-DA fluorescent signal. DCF-DA fluorescent signal is represented as fold change of DCF mean fluorescent intensity (MFI), normalized on cells incubated in growth medium alone. Oxidized BODIPY C11 fluorescent signal represented as MFI fold change normalized on cells incubated in growth medium alone, following 4 hrs incubation with **C** 100 µM or **D** 500 µM of tBHP. Right: FACS analysis of lipid peroxidation following 4 hrs incubation with 100 µM of tBHP, indicated by oxidized BODIPY 581/591 fluorescent signal. **E** Representative images of cell phenotypes following 4 hrs incubation with 100 µM of tBHP, with or without 100 µM β-Mercaptoethanol. **F** Western blot analysis of xCT expression in parental 4T1 cells and several xCT-KO clones, ten of which are used as a pool in *in vivo* experiments. Vinculin is used as loading control. **G** Left: Representative images migrating cells in a transwell migration assay. Right: Percentage of transwell area covered by migrated cells. **H** *Slc7a11* transcript expression levels in 4T1 cells transduced with an empty (4T1-pLVX) or xCT-expressing (4T1-xCT) lentiviral vector, represented as fold change normalized on *Slc7a11* transcript levels in parental 4T1 cells (value = 1). **I** Left: Representative images migrating cells in a transwell migration assay. Right: Percentage of transwell area covered by migrated cells. **J** Cell proliferation curves of parental 4T1-pLVX cells and 4T1-xCT cells, assessed by MTT assay. Optical Densities (O.D.) of dissolved MTT crystals are used as a readout of live cells. Number of replicates: in all presented panels depicting bar graphs, each dot represents an independent biological replicate. Each dot depicting a biological replicate is the result of at least two technical replicates, except for experiments of flow cytometry, where only a technical replicate for biological sample was performed. Images depict representative experiments. Statistical analysis: unpaired t test (panels G, I, J). For experiments that showed high inter-experimental variability of output signal such that normalization on an internal control was required (panels B, C, D), a ratio paired t test was used to test that ratios between the coupled values analyzed are consistent along the experiments. * *p*<0.05; Where not indicated, *p* value is not significant (except in panels A and H where statistical analysis was not performed, as the experiments were performed only once, even though in technical triplicates). Histograms represent mean values. Error bars are shown only when n > 5, and represent SD.


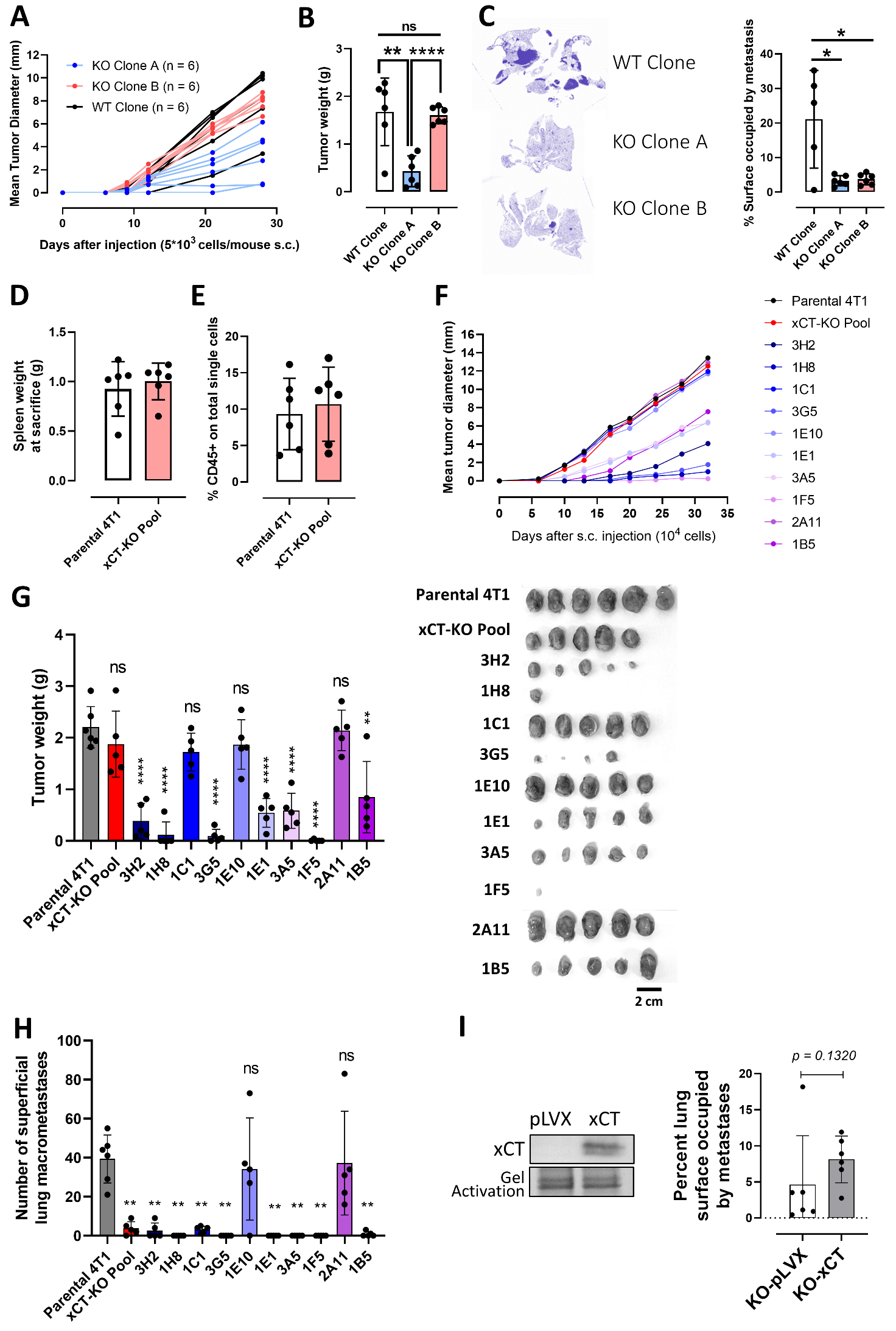


Fig. S5. Effects of xCT modulation in cancer cells on tumor growth and metastasis formation.

**A** Growth curves of tumors deriving from 5x10^3^ 4T1 (WT Clone or KO Clones A and B) injected s.c. in the flank of female BALB/c mice (6 mice per group). Each dot represents a mouse. **B** Histogram representation of tumor weight at sacrifice (derived from mice represented in panel A). **C** Left: representative slices of FFPE lungs (derived from mice represented in panel B) stained with H&E (only hematoxylin channel is displayed to highlight metastases). Right: percentage of lung slice area occupied by metastases. **D** Spleen weight at sacrifice, from mice represented in the Fig. 3B. **E** Percentage of CD45+ tumor-infiltrating cells on total single cells deriving from tumor dissociation (from mice represented in the Fig. 5B), as assessed by flow cytometry. **F** Growth curves of tumors deriving from 10^4^ parental 4T1 cells, xCT^KO^ pool, or single xCT^KO^ clones, injected s.c. in the flank of female BALB/c mice (5 mice per group, except for parental 4T1 cells where 6 mice were used). **G** Left panel: Histogram representation of tumor weight at sacrifice (derived from mice represented in panel F); Right panel: picture of tumors resected from mice. **H** Number of metastatic nodules in the lungs from mice described in panels F and G. **I** Left panel: Western Blot showing xCT re-expression in xCT^KO^ cells transduced with xCT-expressing lentivirus (xCT) as compared to xCT^KO^ cells transduced with a control lentiviral vector (pLVX); Right panel: Lung surface occupied by metastasis in BALB/c mice challenged with a subcutaneous injection of 10^4^ cells. Number of replicates: in panels A-E and G-I each dot represents a mouse. In panel F each dot represents the mean value, and error bars are omitted for better visualization. Statistical analysis: unpaired t test, except for statistical analysis on metastasis where Mann-Whitney test was used. * *p*<0.05; ** *p*<0.01; *** *p*<0.001. In panels G and H, all comparisons are made on the parental 4T1 cells group. Where not indicated, *p* value is not significant (except in panels A and F, where statistical analysis is not reported for a better visualization, and differences in tumor growth are highlighted in panel B and G, respectively). Histograms represent mean values. Error bars represent SD.


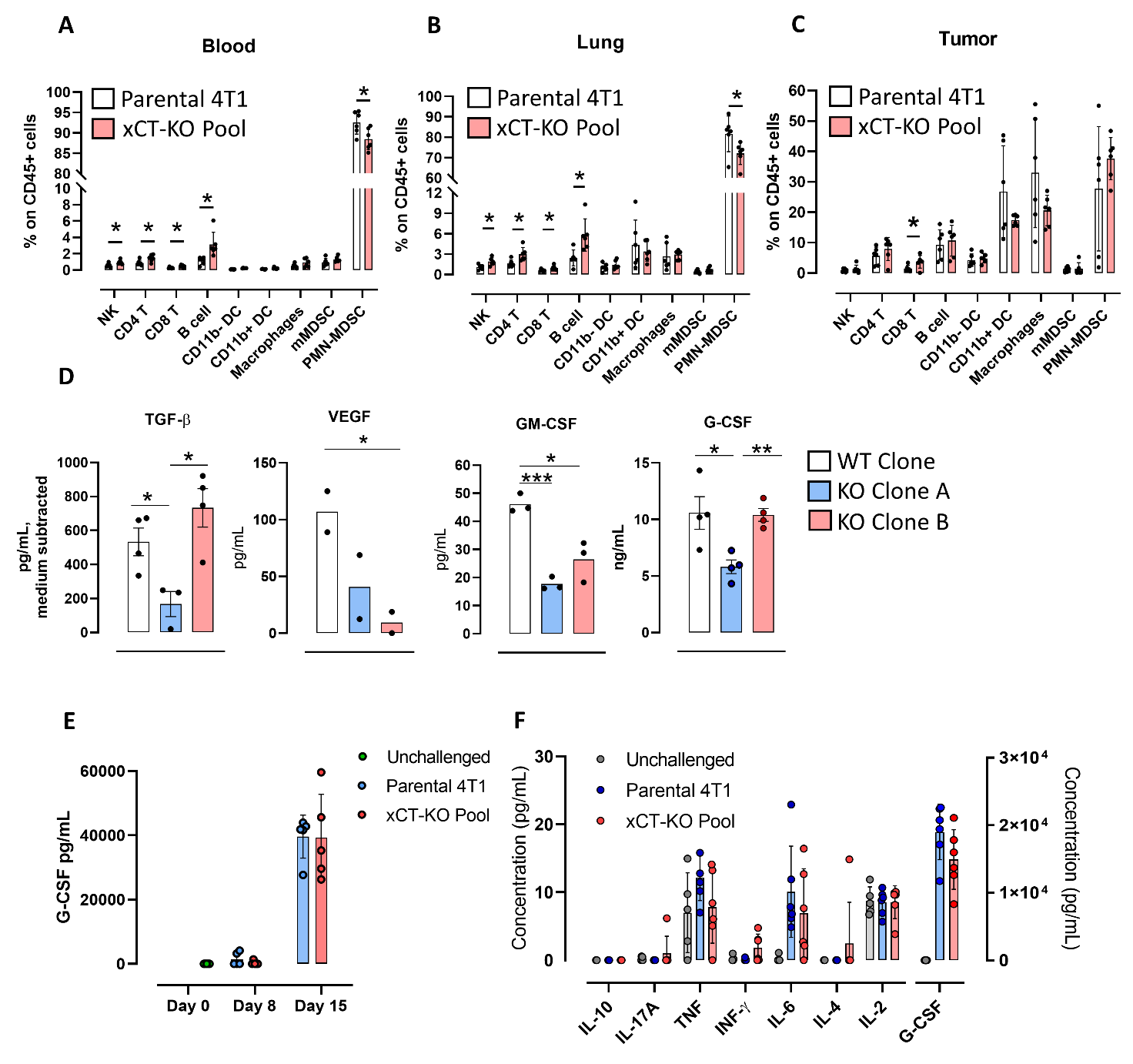


Fig. S6. Effects of the modulation of xCT in cancer cells on the recruitment and polarization of immune cells.

Leukocyte populations proportions on total leukocytes (CD45+) isolated from **A** blood, **B** lung or **C** tumor of mice represented in Fig.3B. **D** Supernatant concentrations of the indicated cytokine, assessed by ELISA. **E** G-CSF concentration in plasma of healthy mice, parental 4T1- and xCT-KO 4T1-bearing mice at days 8 and 15 post challenge. **F** BD Cytometric Bead Array (CBA) Mouse Th1/Th2/Th17 Cytokine analysis to measure cytokines concentration and ELISA to measure G-CSF concentration in plasma of healthy mice, parental 4T1- and xCT-KO 4T1-bearing mice at 30 days post challenge. Number of replicates: in panels A-C and E, F, each dot represents a mouse. In panel D each dot represents an independent biological replicate, each resulting from at least a technical duplicate. Statistical analysis: unpaired t test. * *p*<0.05; ** *p*<0.01; *** *p*<0.001. Where not indicated, *p* value is not significant. Histograms represent mean values. Error bars represent SD.
