# Supplementary material for: Cystine/glutamate antiporter xCT deficiency reduces metastasis without impairing immune system function in breast cancer mouse models
Source: J Exp Clin Cancer Res. 2023 Sep 29;42:254. doi: 10.1186/s13046-023-02830-x (PMC10540318; doi:10.1186/s13046-023-02830-x)
Supplement: Supplementary file 2 — Additional file 2. Supplementary Methods; Key resources table; SI References. [file 13046_2023_2830_MOESM2_ESM.docx]

**Supplementary Methods, Key Resources Table, and SI References from**

**Cystine/glutamate antiporter xCT deficiency reduces metastasis without impairing immune system function in breast cancer mouse models**

Roberto Ruiu^1^, Chiara Cossu^1^, Antonella Iacoviello^1^, Laura Conti^1^, Elisabetta Bolli^1^, Luca Ponzone^2^, Jolanda Magri^1^, Alekya Rumandla^1^, Enzo Calautti^2^, Federica Cavallo^1^

*^1^Laboratory of Oncoimmunology, Molecular Biotechnology Center “Guido Tarone”, Department of Molecular Biotechnology and Health Sciences, University of Turin, Turin, Italy*

*^2^Laboratory of Epithelial Stem Cell Biology and Signaling, Molecular Biotechnology Center “Guido Tarone”, Department of Molecular Biotechnology and Health Sciences, University of Turin, Turin, Italy*

Corresponding author

Federica Cavallo, Molecular Biotechnology Center “Guido Tarone”, Via Nizza, 52, Turin 10126, Italy. Phone: +39 011 670 6457; Fax: +39 011 236 6457; E-mail: [federica.cavallo@unito.it](mailto:federica.cavallo@unito.it)

SUPPLEMENTARY METHODS

**Generation of SUT32-2H9 and WT27 cell lines**

To obtain primary cancer cells from BALB-neuT/xCT^null^ mice (named SUT32-2H9 and WT27 cells, respectively), tumors were minced, digested with 100 µg/mL collagenase (Sigma-Aldrich) in DMEM at 37 °C for 45 min, and passed through a 70 µm cell strainer. Cells were then suspended in DMEM-F12 medium (Sigma-Aldrich) with 20 ng/mL basic fibroblast growth factor (bFGF, PeProtech), 20 ng/mL epidermal growth factor (EGF, Sigma-Aldrich), 5 μg/mL insulin (Sigma-Aldrich), 0.4% bovine serum albumin (BSA, Sigma-Aldrich) and 100 µM β-ME in ultra-low attachment flasks (Corning) to remove contaminating fibroblasts. Medium was refreshed twice per week, and after one week non-adherent tumor spheroids were dissociated using trypsin-EDTA 0.5% (Gibco) at 1X dilution in PBS and re-plated under the same conditions. Upon the subsequent passage, single cells were resuspended in DMEM-F12 medium with 20% FBS and 100 µM β-ME and plated in standard adherent conditions. All growth media used were supplemented with Penicillin/Streptomycin solution (P/S, Sigma-Aldrich) at 1:100 dilution unless otherwise specified. When sub-confluency was reached (approximately every second day) cells were dissociated using trypsin-EDTA 0.5% at 1X dilution in PBS and seeded into new flasks.

**BALB/c-xCT^null^ and BALB-neuT/xCT^null^ mice generation and genotyping**

C3H/HeSnJ-*Slc7a11^sut^*/J (xCT^null^) mice were outcrossed with BALB/c mice and backcrossed for 10 generations with BALB/c mice to obtain xCT^null^ BALB/c mice. xCT^null^ BALB/c females were crossed with male BALB-neuT mice and then with the resulting BALB-neuT/xCT^Het^ male mice to generate a mammary cancer-prone BALB-neuT/xCT^null^ progeny. Genotyping was performed by PCR (reagents from Promega). neuT transgene was assessed as described in Ref.(1). Wild type or mutated (*sut*) form of *Slc7a11* was assessed as described in Ref.(2), except for the *sut* reverse primer that was designed by us: *5’ TTCCTTTTCTGGTGGGTAGTAGG 3’.*

**CRISPR/Cas9 vector production**

Oligonucleotides containing the guide sequences were designed to target the exon 1 of murine *Slc7a11* gene using IDT software (IDT) and verified through the UCSC Genome Browser on Mouse Dec. 2011 (GRCm38/mm10) Assembly.

The following oligonucleotides were synthesized by Thermo Fisher Scientific:

Forward: *5’ CACCGGTTGTGGCCACCATCTCCAA 3’*

Reverse: *5’ AAACTTGGAGATGGTGGCCACAACC 3’*

The oligonucleotides were phosphorylated and annealed using ATP-containing T4 Ligation Buffer (New England Biolabs) in the presence of T4 Polynucleotide Kinase (New England Biolabs). pSpCas9(BB)-2A-Puro (px459) V2.0 plasmid vector was linearized with BbsI (New England Biolabs). The linearized vector and the annealed oligo pair were ligated using T4 Ligation Buffer (New England Biolabs) in the presence of T4 Ligase (New England Biolabs).

DH5α competent *E.coli* (Thermo Fisher Scientific) were transformed with the ligation product through heat shock and then plated on ampicillin-containing agarose plates and incubated overnight at 37 °C. Colonies were picked the following day, expanded in ampicillin-containing lysogeny broth (LB). Plasmid DNA was extracted using MIDI-prep (Qiagen) according to the manufacturer's instructions. Insertion was confirmed through digestion with AgeI (New England Biolabs) and by Sanger sequencing (Service provided by BMR genomics) using a hU6 promoter-specific primer. The vector carrying the *Slc7a11* exon 1-targeting guides is referred to as px459-xCT.

**xCT-overexpressing vector production**

The protein-coding sequence of murine xCT (NM_011990.2 → NP_036120.1) was amplified from xCT-pVAX1 vector (3) via PCR and subcloned into the pLVX-PURO vector. The following primers were used for xCT sequence amplification:

- Forward primer (EcoRI): 5’ CTAGGAATTCGCCGCCACCATGGTCAGAAAGCCAG 3’
- Reverse primer (XbaI): 5’CCGGTCTAGACGTCATAATTCTTTAGAGTCTTCTGGTAC 3’

Following amplification, PCR product was purified using the QIAquick PCR Purification Kit (Qiagen) according to manufacturer’s instructions. 5 μg of purified PCR product and pLVX-PURO empty vector were digested with EcoRI and XbaI (both from New England Biolabs) to obtain complementary sticky ends. After purification using QIAquick PCR Purification Kit (Qiagen), the vector was treated with Calf Intestinal Alkaline Phosphatase (CIAP, Promega) to avoid self-ligation and then purified using the QIAquick PCR Purification Kit. Ligation reaction was performed between the insert and the vector overnight at 4 °C and then at RT for 6 hrs using T4 Ligase (Promega) following manufacturer’s instructions. Competent *E. Coli* bacteria (DH5α, Thermo Fisher Scientific) were then transformed by heat shock and plated on ampicillin-containing agarose plates overnight at 37 °C to select colonies in which correct ligation had occurred through ampicillin resistance. Colonies were expanded and bacterial preparation of samples was performed through QIAprep Spin Miniprep Kit (Qiagen) to obtain enough DNA for downstream applications. Finally, mini prep products were sequenced (BMR Genomics) and digested with XbaI and EcoRI (New England Biolabs) to confirm that xCT coding sequence was inserted in the right orientation within pLVX-Puro vector to obtain xCT-pLVX vector.

**Lentiviral vector Production**

To produce lentiviral vectors for xCT overexpression, 3 x 10^6^ early passage HEK-293T cells were plated in a 10 cm poly-D-lysine Hydrobromide (0.1 mg/mL, Sigma-Aldrich)-coated cell culture dish in DMEM supplemented with 10% FBS without penicillin/streptomycin. 24 hrs post-plating, cells were incubated overnight with transfection reaction consisting of Lipofectamine 2000 (Sigma-Aldrich) and DNA plasmids, both diluted in Opti-MEM (Thermo Fisher Scientific). The following plasmids were used: pCMV-VSV-G (envelope plasmid), pCMV-dR8.74 (packaging plasmid), and empty pLVX-Puro or xCT-pLVX (transfer plasmid). Following overnight incubation, supernatant was removed and substituted with fresh medium. Lentivirus-containing supernatant was collected 48 hrs after, aliquoted and stored at -80 °C until use.

**Transduction of cells with lentiviral vectors (extended)**

Target cells (SUT32-2H9 or 4T1, approx. 30% confluent) were incubated with lentiviral vector-containing supernatant supplemented with Sequa-brene (Sigma-Aldrich) at a final concentration of 8 μg/mL. After 20 h incubation, lentiviral vector-containing supernatant was substituted with fresh growth medium, and the following day selection antibiotic was added (Puromycin, 3 μg/mL for 4T1, 1.5 μg/mL for SUT32-2H9). After 2 days of selection, non-transduced control cells were all dead. Transduced cells were cultured and expanded under continuous antibiotic selection and xCT overexpression was confirmed by real-time PCR or Western Blotting.

**Real-time PCR**

Total RNA was isolated using the Qiazol reagent (Qiagen) following the manufacturer’s instructions. The DNA-free kit (AM1906, Thermo Fisher Scientific) was used to remove genomic DNA contaminations. RNA concentration and quality were estimated with the Nanodrop 2000 spectrophotometer (Thermo Fisher Scientific) and Agilent 2100 Bioanalyzer (Agilent Technologies), respectively. mRNA was retrotranscribed using a High-Capacity cDNA Reverse Transcription Kit (Thermo Fisher Scientific). Target cDNA was amplified through real-time PCR using gene-speciﬁc primers (Mm_Slc7a11_1_SG and Mm_Gapdh_3_SG, QuantiTect Primer Assay, Qiagen) and SYBR Green PCR Master Mix (Applied Biosystems). A 7300 RT-PCR system (Applied Biosystems) and the Applied Biosystems SDS Software Version 1.3.1 were used to perform the real-time PCR and data analysis, respectively. GAPDH expression was used for quantitative normalization. The expression levels relative to control cells were calculated using the comparative ΔΔCt method.

**Selenocystine uptake assay**

The following protocol is an adaptation from (4). Cells were plated in a 6-well plate at a total cell density of 5.8 × 10^5^ cells/well (4T1, in complete RPMI supplemented with 10% FBS and P/S) or 1.2 × 10^6^ cells/well (SUT32-2H9 cells, in complete DMEM-F12 supplemented with 20% FBS and P/S). The following day, cells were incubated pre-warmed uptake buffer at 37℃ with 5% CO2 in a humid atmosphere for 5 min, washed with uptake buffer twice and incubated with uptake buffer (137 mM choline chloride, 3 mM KCl, 1 mM CaCl2, 1 mM MgCl2, 5 mM D-glucose, 0.7 mM K2HPO4, 10 mM HEPES, pH 7.4) containing seleno-L-cystine (Sigma-Aldrich, final concentration 200 uM) under the same conditions for 1 h, with or without the addition of xCT inhibitors erastin (Selleck Biochem Cat# S7242, 2 μM) or sulfasalazine (Sigma-Aldrich Cat# S0883-10G, 1 mM). Cells were then washed with PBS three times and lysed with 400 μL/well of a methanol: water solution (4:1 ratio) kept at -80 °C. After 10 min incubation at -80 °C, cells were scraped on dry ice, collected in Eppendorf tubes and homogenized by shaking (1400 rpm, 5 min 4 °C) followed by sonication in cold water bath. Lysate was centrifuged at 14000 x g, 5 min at 4 °C to remove debris and protein content quantified through BCA Protein assay kit*.* 50 μL lysate/well was distributed in 96-well plate (black, suitable for fluorescence assays), and 200 μL per well of 1.25X concentrated reaction buffer was added and incubated at 37℃ for 30 min. 1.25X concentrated reaction buffer consisted of 2-(N-Morpholino) ethanesulfonic acid (MES, Sigma-Aldrich)-containing buffer 125 mM in dH_2_O, pH6 + Fluorescein O,O′-diacrylate (FOdA, Sigma-Aldrich) 12,5 µM + Tris(2-carboxyethyl)phosphine hydrochloride (TCEP, Sigma-Aldrich) 250 µM. Fluorescence intensity (Ex: 485 nm; Em: 535 nm) was acquired by a microplate reader (Glomax, Promega). The blank for the assay is measured by the same procedure but without using selenocystine substrate. Fluorescence is normalized on protein content of the lysate and further expressed as % fluorescence emission as compared to untreated parental 4T1 cells.

**Cell viability, ROS content, and lipid peroxidation**

4T1 cells were seeded in growth medium without β-ME and penicillin/streptomycin in 6-well plates. The following day, cells were incubated for 4hrs with either 100 µM or 500 µM *tert*-Butyl hydroperoxide solution (tBHP, Luperox^®^ TBH70X, Sigma-Aldrich) in the presence or not of 100 µM β-ME. WT27 cells and SUT32-2H9 cancer cells were cultured for 72 hrs with or without 100 µM β-ME. At the experimental endpoint, dead cells-containing supernatants were collected and pelleted together with trypsin-dissociated cells. For intracellular ROS content detection, cells were resuspended in DMEM (Sigma Aldrich) and incubated with 10 µM 2’,7’-Dichlorofluorescin diacetate (DCF-DA, Sigma-Aldrich Cat# D6883-250MG) for 30’ at 37 °C. To assess lipid peroxidation, cells were resuspended in Hank’s Balanced Salt Solutions (HBSS, Sigma-Aldrich) and incubated with 5 µM C11-BODIPY (BODIPY 581/591 C11, Thermo Fisher Scientific Cat# D3861) for 15 min at 37 °C. Cells were then pelleted and resuspended in the residual volume after supernatant discard. DAPI staining was used to assess cell viability by flow cytometry using the BD FACSVerse^TM^. Data were analyzed using FlowJo V10 software.

**MTT assay**

Cells were seeded in 96 well-plates and incubated for 24, 48, 72 hrs or up to one week. MTT solution (0.5 mg/mL; Sigma Aldrich) was added and incubated for 4 hrs at 37 °C. The supernatant was removed, and DMSO was added to dissolve the formazan crystals. Absorbance was measured at 570 nm with 655 nm measure (background) subtraction, using the iMark microplate reader (BioRad).

**Colony-forming efficiency assay**

500 cells were seeded in 6 well-plate and incubated for 10 days, in the presence or not of 100 µM β-ME, until single-cell colonies were formed. The cell medium was refreshed every three days. At the endpoint, the cells were fixed with 4% formaldehyde solution (Sigma-Aldrich) and stained with crystal violet aqueous solution (0.1% crystal violet, 20% methanol). Then, wells were washed with deionized H_2_O and let dry. Images of the colonies were acquired using a scanner and the percentage of the occupied area in each well was determined using ImageJ.

**Wound healing assay**

The cells were plated in 12 well-plates, incubated until they reached a full confluence, and then starved overnight in FBS-free growth medium. A pipette tip was used to make the wound. After wound making, cells were maintained in medium with 1% FBS. Images were taken at 0, 24, and 48 hrs after making the wound using an Axio Observer microscope (Zeiss) at a 10X magnification. The percentage of wound closure was determined using ImageJ and calculated as follows:

$$\% Wound Closure t(x) = \frac{Wound Area t(0) - Wound Area t(x)}{Wound Area t(0)}$$

**Transwell migration assay**

Cells were plated on the top of Transwell permeable supports (8 μm polycarbonate membrane, Corning) in serum-free medium and inserted within a 24-well plate containing medium with FBS. After 48 hrs incubation, cells were wiped away from the top side of transwell supports, and cells remaining on the bottom side were fixed in 4% formaldehyde and stained with 0.2% Crystal Violet (Sigma-Aldrich) solution in methanol. Membranes were rinsed in water and air-dried. Pictures of 5 fields covering most of the membrane surface were taken with a BX41 microscope (Olympus Corporation) at 4X magnification. Images were analyzed with ImageJ. For each condition, the average % area occupied by cells in the five fields was calculated.

**Cytokine ELISA**

Supernatants were collected from cells plated at the same density after 48 hrs of culture and centrifuged to remove cell debris. Plasma was harvested from healthy mice and 4T1 parental and 4T1 xCT^KO^ - bearing mice 8, 15 and 30 days after injection and stored at -80 °C until analysis. DuoSet^Ⓡ^ ELISA kits (RnD systems) were used to measure mouse TGF-β (Cat# DY1679-05), VEGF (Cat# DY493-05), GM-CSF (Cat# DY415-05), and G-CSF (Cat# DY414-05) levels, according to the manufacturer’s instructions. To measure the concentration of G-CSF in plasma, samples were further diluted 1:100.

**Cytometric Bead Array Cytokine Analysis**

Plasma harvested from healthy mice, 4T1 parental and 4T1 xCT^KO^ -bearing mice 30 days after injection were tested with BD TM Cytometric Bead Array (CBA) Mouse Th1/Th2/Th17 Cytokine Kit (BD Bioscience Cat# 560485, RRID: AB_2869354) to measure Interleukin-2 (IL-2), Interleukin-4 (IL-4), Interleukin-6 (IL-6), Interferon-γ (IFN-γ), Tumor Necrosis Factor (TNF), Interleukin-17A (IL-17A), and Interleukin-10 (IL-10) protein levels, according to the manufacturer’s instructions.

**Immunophenotyping (extended)**

Erythrocytes were lysed using an erythrocyte lysis buffer (155 mM NH_4_Cl, 15.8 mM Na_2_CO_3_, 1 mM EDTA, pH 7.3) for 10 min then rinsed with PBS and centrifuged. Fc Blocking antibody (anti-CD16/CD32 antibody, BioLegend Cat# 101320) was added, and the cells were stained at 4 °C for 30 min with fluorescent-labeled antibodies. The following antibody combinations were used: CD45 VioGreen (Miltenyi Biotec Cat# 130-123-900), CD3 FITC (Miltenyi Biotec Cat# 130-119-135), CD49b PE (Miltenyi Biotec Cat# 130-123-702), CD4 APC-Vio 770 (Miltenyi Biotec Cat# 130-119-134), CD8 VioBlue (Miltenyi Biotec Cat# 130-123-865), PD1 APC (Miltenyi Biotec Cat# 130-102-263), CD69 PE-Cyanine7 (BioLegend Cat# 104512) for T cells and NK cells; CD45 VioGreen (Miltenyi Biotec Cat# 130-123-900), CD11b FITC (Miltenyi Biotec Cat# 130-113-234), F4/80 PE-Vio 770 (Miltenyi Biotec Cat# 130-118-459), Ly6G VioBlue (Miltenyi Biotec Cat# 130-119-986), Ly6C APC-Vio 770 (Miltenyi Biotec Cat# 130-121-439), MHC II APC (Miltenyi Biotec Cat# 130-102-139), CD206 PE (BioLegend Cat# 141706) for Myeloid-Derived Suppressor Cells and macrophages; CD45 VioGreen (Miltenyi Biotec Cat# 130-123-900), CD11b FITC (Miltenyi Biotec Cat# 130-113-234), CD11c APC (Miltenyi Biotec Cat# 130-119-802), CD45R APC-Vio 770 (Miltenyi Biotec Cat# 130-110-848), I-Ad PE (BD Biosciences Cat# 553548) for granulocytes, dendritic cells, and B cells. Live/dead cells were discriminated by propidium iodide staining. For the characterization of SUT32-2H9 and WT27 cell lines, the following antibodies were used: Rabbit anti-Cytokeratin 14 (CK14) antibody [LL002] (FITC) (Abcam Cat# ab77684); Rabbit anti-Cytokeratin 19 (CK19) antibody (Abcam Cat# ab15463); alpha smooth muscle Actin (αSMA) antibody [1A4] (FITC) (Abcam Cat# ab8211); mouse anti-c-ERBB2/c-Neu (Ab-4) antibody (Millipore Cat# OP16-100UG). The following secondary antibodies were used in combination with unconjugated primary antibodies: Rabbit Anti-Mouse Immunoglobulins/FITC (Dako Cat# F0261); Swine Anti-Rabbit Immunoglobulins/FITC (Dako Cat# F0205).

KEY RESOURCES TABLE

| REAGENT or RESOURCE | SOURCE | IDENTIFIER |
| --- | --- | --- |
| Antibodies | | |
| Rat anti-mouse CD16/CD32 antibody (Fc Blocking) | BioLegend | Cat# 101320; RRID: AB_1574975 |
| Rat anti-mouse CD45 antibody, VioGreen | Miltenyi Biotec | Cat# 130-123-900; RRID: AB_2811572 |
| Rat anti-mouse CD3ε antibody, FITC | Miltenyi Biotec | Cat# 130-119-135; RRID: AB_2751635 |
| Rat anti-mouse CD49b antibody, PE | Miltenyi Biotec | Cat# 130-123-702; RRID:AB_2811545 |
| Rat anti-mouse CD4 antibody, APC-Vio770 | Miltenyi Biotec | Cat# 130-119-134; RRID:AB_2751634 |
| Rat anti-mouse CD8a antibody, VioBlue | Miltenyi Biotec | Cat# 130-123-865; RRID:AB_2811566 |
| Rat anti-mouse CD279 (PD1) antibody, APC | Miltenyi Biotec | Cat# 130-102-263; RRID:AB_2661365 |
| Hamster anti-mouse CD69 antibody, PE/Cyanine7 | BioLegend | Cat# 104512; RRID: AB_493564 |
| Rat anti-mouse/human CD11b antibody, FITC | Miltenyi Biotec | Cat# 130-113-234; RRID:AB_2733615 |
| Recombinant human anti-mouse F4/80 antibody, PE-Vio770 | Miltenyi Biotec | Cat# 130-118-459; RRID:AB_2733260 |
| Recombinant human anti-mouse Ly-6G antibody, VioBlue | Miltenyi Biotec | Cat# 130-119-986; RRID:AB_2751964 |
| Rat anti-mouse Ly-6C antibody, APC-Vio770 | Miltenyi Biotec | Cat# 130-121-439; RRID:AB_2784429 |
| Rat anti-mouse MHC Class II antibody, APC | Miltenyi Biotec | Cat# 130-102-139; RRID:AB_2660058 |
| Rat anti-mouse CD206 antibody, PE | BioLegend | Cat# 141706; RRID:AB_10895754 |
| Hamster anti-mouse CD11c antibody, APC | Miltenyi Biotec | Cat# 130-119-802; RRID:AB_2751854 |
| Recombinant human anti-mouse CD45R (B220) antibody, APC-Vio770 | Miltenyi Biotec | Cat# 130-110-848; RRID:AB_2658284 |
| Mouse anti-mouse anti-I-A[d] antibody, PE | BD Biosciences | Cat# 553548; RRID:AB_394915 |
| Mouse anti-Cytokeratin 14 antibody [LL002], FITC | Abcam | Cat# ab77684; RRID:AB_2265437 |
| Rabbit anti-Cytokeratin 19 antibody | Abcam | Cat# ab15463; RRID:AB_2281021 |
| Mouse anti-Alpha smooth muscle Actin antibody [1A4] | Abcam | Cat# ab8211; RRID:AB_306359 |
| Mouse anti-c-ERBB2/c-Neu antibody (Ab4) | Millipore | Cat# OP16-100UG; RRID:AB_213323 |
| Rabbit anti-mouse immunoglobulins, FITC | Dako | Cat# F0205 |
| Swine anti-rabbit immunoglobulins, FITC | Dako | Cat# F0261 |
| Rabbit anti-mouse xCT/SLC7A11 antibody | Cell Signaling Technology | Cat# 98051; RRID:AB_2800296 |
| Mouse anti-Vinculin antibody | Avalle et al. (5) | N/A |
| Goat anti-mouse IgG, HRP | Sigma-Aldrich | Cat# A4416; RRID:AB_258167 |
| Goat anti-rabbit IgG, HRP | Sigma-Aldrich | Cat# A0545; RRID: AB_257896 |
| Bacterial and virus strains | | |
| *E.coli*: DH5α Competent Cells | Thermo Fisher Scientific | Cat# 18265017 |
| Chemicals, peptides, and recombinant proteins | | |
| RPMI-1640 | Sigma-Aldrich | Cat# R8758 |
| DMEM/F12 | Sigma-Aldrich | Cat# RNBK8509 |
| DMEM | Sigma-Aldrich | Cat# RNBK9599 |
| Opti-MEM | Gibco | Cat# 11058-021 |
| Human Plasma-Like Medium (HPLM) | Gibco | Cat# A4899101 |
| Collagenase | Sigma-Aldrich | Cat# C5138 |
| Trypsin-EDTA Solution 10X | Sigma-Aldrich | Cat# 59418C |
| Puromycin Dihydrochloride | Sigma-Aldrich | Cat# P9620 |
| BSA | Sigma-Aldrich | Cat# A9418 |
| FBS | Sigma-Aldrich | Cat# S0615 |
| Slide-A-Lyzer Dialysis Cassette 3.500 MWCO | Thermo Fisher Scientific | Cat# 66130 |
| EGF | Sigma-Aldrich | Cat# E9644 |
| bFGF | PeProtech | Cat# 100-18B |
| Insulin | Sigma-Aldrich | Cat# I0516 |
| T4 Polynucleotide Kinase | New England Biolabs | Cat# M0201S |
| T4 Ligase + T4 Ligation Buffer | Promega | Cat# M1801 |
| T4 Ligase + T4 Ligation Buffer | New England Biolabs | Cat# M0202 |
| BbsI | New England Biolabs | Cat# R0539S |
| AgeI | New England Biolabs | Cat# R0552 |
| EcoRI | New England Biolabs | Cat# R0101L |
| XbaI | New England Biolabs | Cat# R0145L |
| Calf Intestinal Alkaline Phosphatase (CIAP) | Promega | Cat# M1821 |
| Lipofectamine 2000 | Thermo Fisher Scientific | Cat# 11668-019 |
| Sequabrene | Sigma-Aldrich | Cat# S2667 |
| Poly-D-Lysine Hydrobromide | Sigma-Aldrich | Cat# P6407 |
| Protease inhibitor cocktail | Sigma-Aldrich | Cat# P8340 |
| Β-Mercaptoethanol | Sigma-Aldrich | Cat# M7522 |
| ECL Substrate | Cyanagen | Cat# XLS142,0250 |
| Seleno-L-cystine | Sigma-Aldrich | Cat# 545996 |
| 2-(N-Morpholino) ethanesulfonic acid (MES) | Sigma-Aldrich | Cat# M3671 |
| Fluorescein O,O′-diacrylate (FOdA) | Sigma-Aldrich | Cat# 570257 |
| Tris(2-carboxyethyl)phosphine hydrochloride (TCEP) | Sigma-Aldrich | Cat# C4706 |
| Erastin | Selleck Biochem | Cat# S7242 |
| Sulfasalazine | Sigma-Aldrich | Cat# S0883 |
| *tert*-Butyl hydroperoxide solution 70 wt. in H2O | Sigma-Aldrich | Cat# 458139 |
| 2’,7’-Dichlorofluorescin diacetate (DCF-DA) | Sigma-Aldrich | Cat# D6883 |
| BODIPY 581/591 C11 | Thermo Fisher Scientific | Cat# D3861 |
| Thiazolyl Blue Tetrazolium Bromide (MTT) | Sigma-Aldrich | Cat# M5655 |
| Carboxyfluorescein succinimidyl ester (CSFE) | Thermo Fisher Scientific | Cat# V12883 |
| Immunodominant rat ERBB2 (p185neu [63–71] 9-mer) peptide: TYVPANASL | Quaglino et al. (6) | N/A |
| Her2/ERBB2 Protein, Rat, Recombinant (ECD, His Tag) | Sino Biological | Cat# 80079-R08H |
| Her2/ERBB2 Protein, Human, Recombinant (ECD, His Tag) | Sino Biological | Cat# 10004-H08H |
| Critical commercial assays | | |
| GoTaq® G2 Flexi DNA Polymerase Kit | Promega | Cat# M7805 |
| MycoAlert^TM^ Mycoplasma Detection Kit | Lonza | Cat# LT07-318 |
| QIAquick PCR Purification Kit | Qiagen | Cat# 28104 |
| QIAprep Spin Miniprep Kit | Qiagen | Cat# 27104 |
| DNA-*free*™ DNA Removal Kit | Thermo Fisher Scientific | Cat# AM1906 |
| High-Capacity cDNA Reverse Transcription Kit | Thermo Fisher Scientific | Cat# 4368814 |
| SYBR™ Green PCR Master Mix | Applied Biosystems | Cat# 4309155 |
| Mm_Slc7a11_1_SG QuantiTect Primer Assay | Qiagen | Cat# 249900; GeneGlobe ID: QT00155757 |
| Mm_Gapdh_3_SG QuantiTect Primer Assay | Qiagen | Cat# 249900; GeneGlobe ID: QT01658692 |
| BD Cytometric Bead Array (CBA) Mouse Th1/Th2/Th17 Cytokine Kit | BD Biosciences | Cat# 560485; RRID: AB_2869354 |
| TGF-β DuoSet^Ⓡ^ ELISA kit | RnD Systems | Cat# DY1679-05 |
| VEGF DuoSet^Ⓡ^ ELISA kit | RnD Systems | Cat# DY493-05 |
| GM-CSF DuoSet^Ⓡ^ ELISA kit | RnD Systems | Cat# DY415-05 |
| G-CSF DuoSet^Ⓡ^ ELISA kit | RnD Systems | Cat# DY414-05 |
| BCA Protein Assay Kit | Thermo Fisher Scientific | Cat# 23225 |
| Experimental models: Cell lines | | |
| Mouse: 4T1 | ATCC | Cat# CRL-2539; RRID: CVCL_0125 |
| Mouse: 4T1-pLVX | This paper | N/A |
| Mouse: 4T1-xCT (xCT-overexpressing) | This paper | N/A |
| Mouse: 4T1 xCT^WT^ Clone (D2) | This paper | N/A |
| Mouse: 4T1 xCT^KO^ Clone A (A2xd12) | This paper | N/A |
| Mouse: 4T1 xCT^KO^ Clone B (B2zd11) | This paper | N/A |
| Mouse: 4T1 xCT^KO^ pool (10 clones) | This paper | N/A |
| Mouse: WT27 | This paper | N/A |
| Mouse: SUT32-2H9 | This paper | N/A |
| Mouse: SUT32-2H9-pLVX | This paper | N/A |
| Mouse: SUT32-2H9-xCT (xCT-expressing) | This paper | N/A |
| Experimental models: Organisms/strains | | |
| Mouse: C3H/HeSnJ-*Slc7a11^sut^*/J (xCT^null^) | Jackson Laboratory | RRID:IMSR_JAX:001310 |
| Mouse: BALB/c | Internal breeding at Molecular Biotchnology Center, Turin, Italy | N/A |
| Mouse: BALB/c-*Slc7a11^sut^* (BALB/c-xCT^null^) | This paper | N/A |
| Mouse: BALB-neuT | Internal breeding at Molecular Biotchnology Center, Turin, Italy, Boggio et al. (7) | N/A |
| Mouse: BALB-neuT/*Slc7a11^sut^* (BALB-neuT/xCT^null^) | This paper | N/A |
| Oligonucleotides | | |
| Fwd primer for NeuT: ATCGGTGATGTCGGCGATAT | Lollini et al. (1) | N/A |
| Rev primer for NeuT: GTAACACAGGCAGATGTAGGA | Lollini et al. (1) | N/A |
| Fwd primer for Slc7a11^wt^: GAAGTGCTCCGTGAAGAAGG | Jackman et al. (2) | N/A |
| Rev primer for Slc7a11^wt^: ATCTCAATCCTGGGCAGATG | Jackman et al. (2) | N/A |
| Fwd primer for Slc7a11^sut^: CCACTGTTGTAGGTCAGCTTAGG | Jackman et al. (2) | N/A |
| Rev primer for Slc7a11^sut^: TTCCTTTTCTGGTGGGTAGTAGG | This paper | N/A |
| Fwd guide sequence for CRISPR/Cas9 (Mouse *Slc7a11*, exon 1): CACCGGTTGTGGCCACCATCTCCAA | This paper | N/A |
| Rev guide sequence for CRISPR/Cas9 (Mouse *Slc7a11*, exon 1): AAACTTGGAGATGGTGGCCACAACC | This paper | N/A |
| Fwd primer for Slc7a11 subcloning (EcoRI): CTAGGAATTCGCCGCCACCATGGTCAGAAAGCCAG | This paper | N/A |
| Rev primer for Slc7a11 subcloning (XbaI): CCGGTCTAGACGTCATAATTCTTTAGAGTCTTCTGGTAC | This paper | N/A |
| Recombinant DNA | | |
| CRISPR/Cas9 Vector: pSpCas9(BB)-2A-Puro (px459) V2.0 | Feng Zahng Lab, Broad Institute, USA | Addgene plasmid # 62988; RRID:Addgene_62988 |
| Donator plasmid: pVAX1-xCT | Lanzardo et al. (3) | N/A |
| Transfer plasmid: pLVX-Puro | Clonetech | Cat# 632159 |
| Transfer plasmid: pLVX-xCT | This paper | N/A |
| Envelope plasmid: pCMV-VSV-G | Robert Weinberg Lab, Whitehead Institute for Biomedical Research, USA | Addgene plasmid # 8454; RRID:Addgene_8454 |
| Packaging plasmid: pCMV-dR8.74 | Didier Trono Lab, EPFL, Switzerland | Addgene plasmid # 22036; RRID:Addgene_22036 |
| Control vaccination DNA plasmid: pVAX1 | Thermo Fisher Scientific | Cat# V26020 |
| Anti-chimeric Rat/Human ERBB2 vaccination DNA plasmid: RHuT | Quaglino et al. (6) | N/A |
| Software and algorithms | | |
| SDS Software V1.3.1 | Applied Biosystems | RRID:SCR_015806 |
| FlowJo V10 | BD Bioscience | RRID:SCR_008520 |
| ImageJ | NIH | RRID:SCR_003070 |
| GraphPad Prism V8 | Dotmatics | RRID:SCR_002798 |
| CRISPR Genome Editing Tool | IDT Integrated DNA Technologies | [Custom Alt-R® CRISPR-Cas9 guide RNA \| IDT (idtdna.com)](https://eu.idtdna.com/site/order/designtool/index/CRISPR_CUSTOM) |
| Other | | |
| Sequencing service | BMR Genomics, Padova, Italy | N/A |

SI REFERENCES

1. Lollini PL, Nicoletti G, Landuzzi L, De Giovanni C, Rossi I, Di Carlo E, et al. Down regulation of major histocompatibility complex class I expression in mammary carcinoma of HER-2/neu transgenic mice. Int J Cancer. 1998;77(6).

2. Jackman NA, Melchior SE, Hewett JA, Hewett SJ. Non-cell autonomous influence of the astrocyte system X-c on hypoglycaemic neuronal cell death. ASN Neuro. 2012;4(1).

3. Lanzardo S, Conti L, Rooke R, Ruiu R, Accart N, Bolli E, et al. Immunotargeting of antigen xCT attenuates stem-like cell behavior and metastatic progression in breast cancer. Cancer Res. 2016;76(1):62–72.

4. Shimomura T, Hirakawa N, Ohuchi Y, Ishiyama M, Shiga M, Ueno Y. Simple Fluorescence Assay for Cystine Uptake via the xCT in Cells Using Selenocystine and a Fluorescent Probe. 2021;

5. Avalle L, Raggi L, Monteleone E, Savino A, Viavattene D, Statello L, et al. STAT3 induces breast cancer growth via ANGPTL4, MMP13 and STC1 secretion by cancer associated fibroblasts. Oncogene 2022 4110. 2022 Jan 18;41(10):1456–67.

6. Quaglino E, Mastini C, Amici A, Marchini C, lezzi M, Lanzardo S, et al. A better immune reaction to Erbb-2 tumors is elicited in mice by DNA vaccines encoding rat/human chimeric proteins. Cancer Res. 2010;70(7):2604–12.

7. Boggio K, Nicoletti G, Carlo E Di, Cavallo F, Landuzzi L, Melani C, et al. Interleukin 12-mediated Prevention of Spontaneous Mammary Adenocarcinomas in Two Lines of Her-2/ neu Transgenic Mice. J Exp Med. 1998;188(3):589–96.
