# Supplementary material for: Cystine/glutamate antiporter xCT deficiency reduces metastasis without impairing immune system function in breast cancer mouse models
Source: J Exp Clin Cancer Res. 2023 Sep 29;42:254. doi: 10.1186/s13046-023-02830-x (PMC10540318; doi:10.1186/s13046-023-02830-x)
Supplement: Supplementary file 3 — Additional file 3. Uncropped western blots. [file 13046_2023_2830_MOESM3_ESM.docx]

**Uncropped Western Blots from**

**Cystine/glutamate antiporter xCT deficiency reduces metastasis without impairing immune system function in breast cancer mouse models**

Roberto Ruiu^1^, Chiara Cossu^1^, Antonella Iacoviello^1^, Laura Conti^1^, Elisabetta Bolli^1^, Luca Ponzone^2^, Jolanda Magri^1^, Alekya Rumandla^1^, Enzo Calautti^2^, Federica Cavallo^1^

*^1^Laboratory of Oncoimmunology, Molecular Biotechnology Center “Guido Tarone”, Department of Molecular Biotechnology and Health Sciences, University of Turin, Turin, Italy*

*^2^Laboratory of Epithelial Stem Cell Biology and Signaling, Molecular Biotechnology Center “Guido Tarone”, Department of Molecular Biotechnology and Health Sciences, University of Turin, Turin, Italy*

Corresponding author

Federica Cavallo, Molecular Biotechnology Center “Guido Tarone”, Via Nizza, 52, Turin 10126, Italy. Phone: +39 011 670 6457; Fax: +39 011 236 6457; E-mail: [federica.cavallo@unito.it](mailto:federica.cavallo@unito.it)

**Uncropped version of Western Blot depicted in Fig.4A**

**
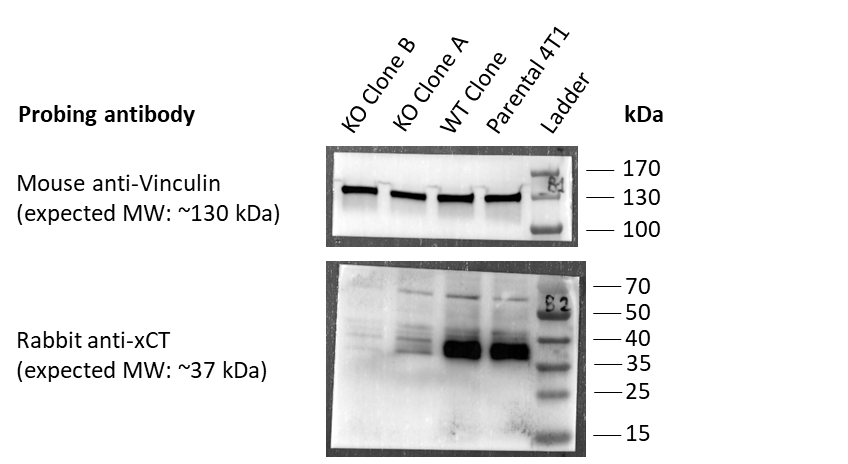
**

The membrane was sliced horizontally between 70 and 100 kDa. The upper membrane was probed with mouse anti-Vinculin antibody, the lower membrane was probed with rabbit anti-xCT antibody. Chemiluminescence and colorimetric images are merged to better visualize the specific bands in relation to the ladder (Thermo Fisher Scientific Cat# 26616). The cropped version represented in Fig.4A is rotated horizontally as compared to the original uncropped version. Experimental details on western blots and antibodies used are reported in the Materials & Methods section.

**Uncropped version of Western Blot depicted in Fig.5A**

**
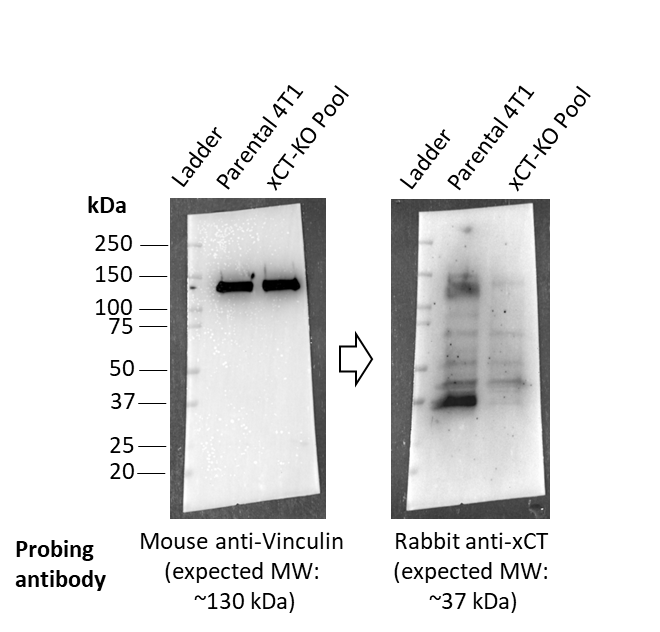
**

The whole membrane was probed with mouse anti-vinculin antibody, then reprobed with rabbit anti-xCT antibody. Chemiluminescence and colorimetric images are merged to better visualize the specific bands in relation to the ladder (Bio-Rad Cat# 1610374). The additional intense band that can be seen just above 100 kDa in the anti-xCT antibody-probed membrane is likely due to an incomplete separation of xCT from its chaperone protein SLC3A2, which is bound to xCT through a disulphide bond. Experimental details on western blots and antibodies used are reported in the Materials & Methods section.

**Uncropped version of Western Blot depicted in Supplementary Fig.4F**

**
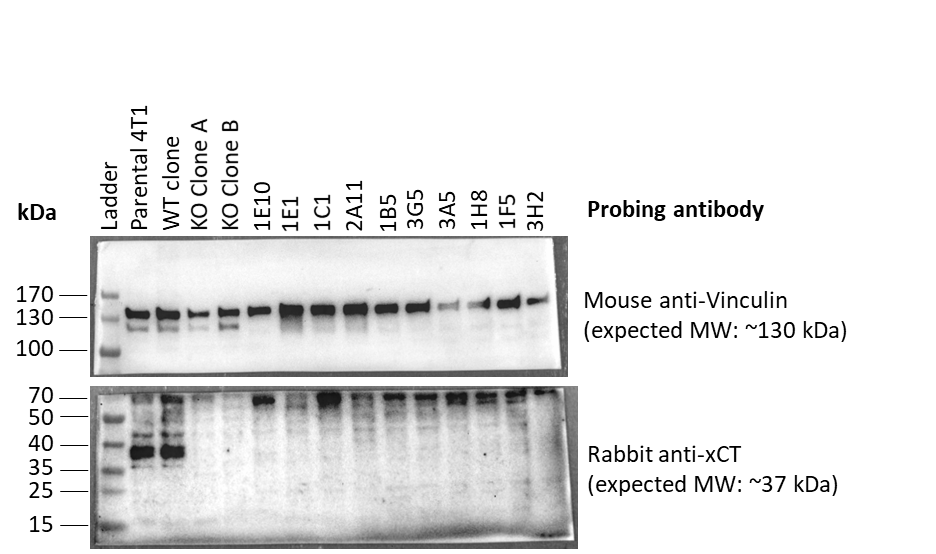
**

The membrane was sliced horizontally between 70 and 100 kDa. The upper membrane was probed with mouse anti-Vinculin antibody, the lower membrane was probed with rabbit anti-xCT antibody. Chemiluminescence and colorimetric images are merged to better visualize the specific bands in relation to the ladder (Thermo Fisher Scientific Cat# 26616). Experimental details on western blots and antibodies used are reported in the Materials & Methods section.

**Uncropped version of Western Blot depicted in Supplementary Fig.5F**


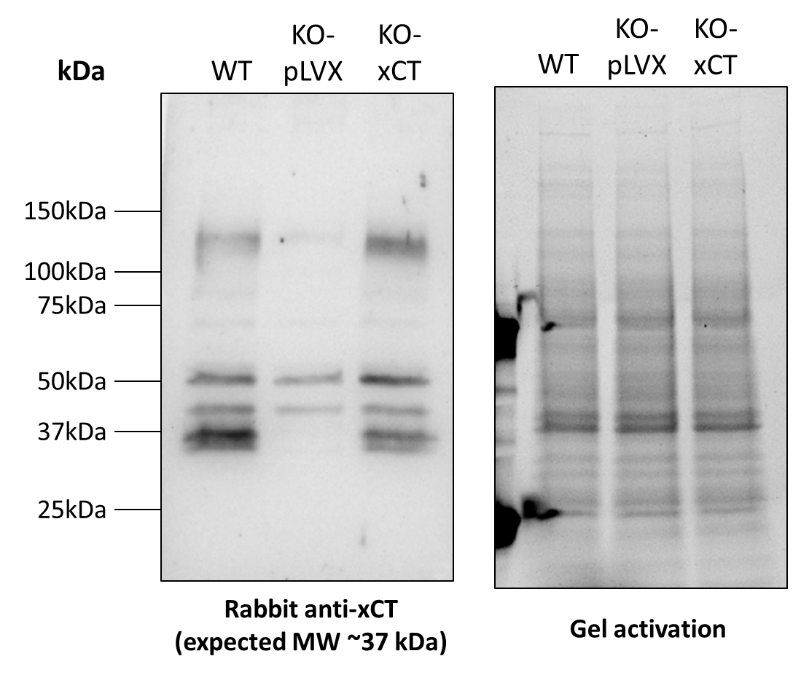
 The whole membrane was probed with rabbit anti-xCT antibody. Gel-activation of the stain-free gel was performed to ensure even protein loading into wells. KO-pLVX and KO-xCT refer to 4T1 xCT-KO pool transduced with empty lentiviral vector (pLVX) or xCT-expressing vector (xCT), respectively. Parental 4T1 cells (WT) were used as control.
